# Methylated GRP94 promotes liver fibrosis progression by enhancing endoplasmic reticulum stress

**DOI:** 10.1038/s41419-026-09159-1

**Published:** 2026-08-05

**Authors:** Xinyi Liu, Kejia Lu, Zitao Jiao, Yahui Zou, Hui Li, Wei Jiang, Xiaowei Zhang

**Affiliations:** Department of Biochemistry and Molecular Biology, School of Basic Medical Sciences, Beijing Key Laboratory of Protein Posttranslational Modifications and Cell Function, Peking University Health Science Center, Beijing, PR China

**Keywords:** Predictive markers, Methylation

## Abstract

Liver fibrosis is a crucial and potentially reversible stage in the progression from chronic liver diseases towards liver cirrhosis and hepatocellular carcinoma (HCC). Endoplasmic reticulum (ER) stress is closely related to hepatic stellate cell (HSC) activation and liver fibrosis. Liver fibrosis is frequently accompanied by a global increase in hepatic protein methylation. This suggests that there may be potential unexplored connections among ER stress, protein methylation, and liver fibrosis. Here, we identify PRMT1-mediated methylation of the ER chaperone GRP94 at arginine 395 as a critical event that drives HSC activation and liver fibrogenesis. Using proteomic profiling of liver tissues from mouse models induced by carbon tetrachloride (CCl_4_) or a choline-deficient, L-amino acid-defined high-fat diet (CDAHFD), combined with studies in human hepatic stellate cells (primary HSCs and the LX2 cell line), we identify that methylation of GRP94 stabilizes the protein by inhibiting its ubiquitination. This modification potentiates the PERK–eIF2α–ATF4 arm of the ER stress response, leading to upregulation of the fibrogenic genes *ACTA2* and *COL1A1* and promoting HSC activation. Pharmacological inhibition of PRMT1 with MS023 reverses GRP94 methylation, suppresses HSC activation, and attenuates fibrosis progression in both cellular systems and murine models. This finding positions the crosstalk between ER stress and protein arginine methylation as a central node in the pathogenesis of liver fibrosis, suggesting that targeting the methylation of GRP94 may be a potential therapeutic strategy of liver fibrosis.

**Figure:**
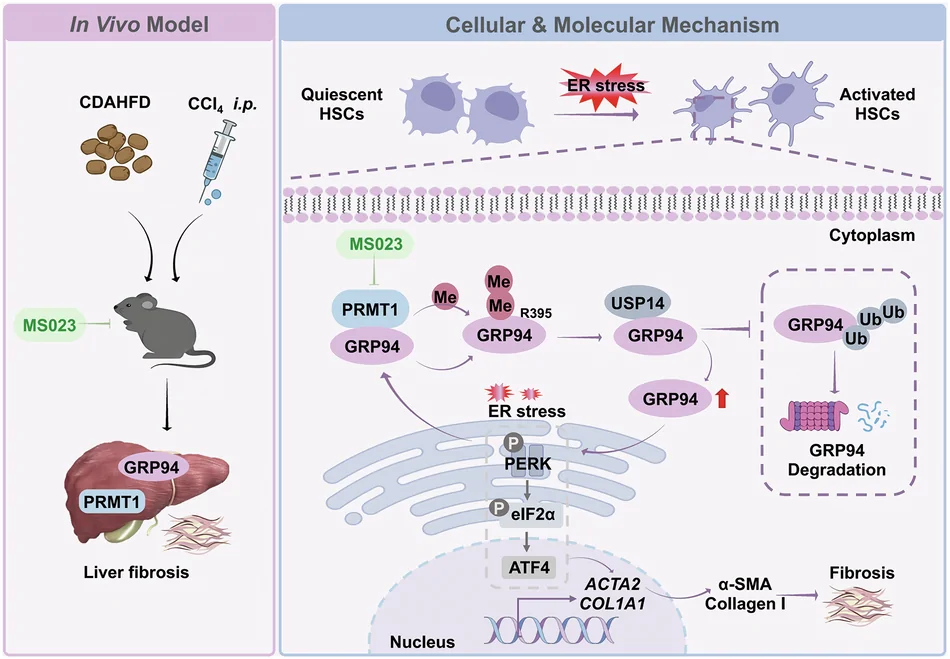

## Introduction

Liver fibrosis represents a critical pathological phase during the progression of various chronic liver disorders. It is characterized primarily by the aberrant proliferation and deposition of hepatic extracellular matrix (ECM), leading to architectural distortion and compromised physiological function of liver tissue [1–4]. It culminates in cirrhosis, portal hypertension, and hepatocellular carcinoma (HCC) [5], causing a massive and growing global health burden [6]. Crucially, however, a critical therapeutic window exists prior to its progression to irreversible cirrhosis, underscoring the imperative to elucidate the underlying mechanisms of its regression [4, 7]. Early-stage liver fibrosis has been confirmed to be reversible, and two key strategies for promoting its regression have been identified: inhibiting hepatocyte apoptosis, which exacerbates liver damage, and promoting the inactivation of hepatic stellate cells (HSCs), which contribute to the accumulation of ECM. The central event in hepatic fibrosis is the activation of quiescent HSCs. In healthy livers, HSCs are quiescent, primarily storing vitamin A. However, chronic liver injury stimulates quiescent HSCs to activate, which then produce abundant ECM components, such as collagen. While the initial triggers of HSC activation are increasingly understood, the intracellular signaling networks and post-translational mechanisms that sustain this pathogenic phenotype remain incompletely elucidated, representing a critical knowledge gap in therapeutic development [7–9].

Endoplasmic reticulum (ER) stress has recently been recognized as a pivotal microenvironmental cue driving HSC activation and fibrogenesis [10–12]. Under the long-term metabolic and inflammatory stress, the disruption of the redox balance within the ER lumen [13] and the accumulation of unfolded/faulty proteins form a vicious cycle, activating the unfolded protein response (UPR) [14, 15]. Although mild ER stress contributes to restoring protein homeostasis through defense mechanisms such as ER-associated degradation (ERAD), UPR, and autophagy, sustained and maladaptive ER stress shifts from pro-survival to pro-inflammatory and pro-fibrotic pathway, activating downstream cascades such as NF-κB and JNK, and upregulating pro-fibrogenic factors like TGF-β1. It leads to hepatocyte death and the activation of Kupffer cells and HSCs, which are strongly associated with liver damage, inflammation, fibrosis, and even HCC [12, 16]. Notably, key ER-resident molecular chaperones, including the 94 kDa glucose-regulated protein (GRP94), are significantly upregulated in both experimental and human liver disease [17]. GRP94, an essential component of the ER quality control system, is traditionally associated with client protein folding and maturation [18, 19]. However, the emerging evidence suggests it may actively participate in signal transduction and cellular responses, for instance, by modulating the maturation and function of Toll-like receptors and integrins, thereby directly impinging on inflammatory and fibrogenic signaling [20]. This raises a compelling hypothesis: GRP94 is not merely a passive marker of ER stress in fibrosis but may be an active, regulated contributor to HSCs pathology. Yet, the precise mechanisms regulating its function, particularly post-translational modifications, in HSCs are entirely unknown.

The role of arginine methylation modification in the malignant progression of benign liver lesions to malignant ones has received increasing attention [21, 22]. As the main enzymes regulating arginine methylation, protein arginine methyltransferases (PRMTs), are the main regulators of signal transduction, stress responses, and gene expression, and play a key role in the pathogenesis of liver diseases [22]. Many PRMTs are abnormally expressed in chronic liver diseases and HCC, promoting epithelial-mesenchymal transition (EMT), and the progression of tumors. PRMT1, the predominant type I enzyme responsible for generating asymmetric dimethylarginine (ADMA) and monomethylated arginine (MMA) formation, has been implicated in various chronic liver diseases [23, 24]. Research indicated that PRMT1 expression is heightened in the liver of cirrhotic individuals and that it significantly contributes to hepatic fibrogenesis [25]. Intriguingly, recent studies have shown a potential nexus between ER stress and arginine methylation, suggesting that PRMT1 exacerbates ER stress by promoting the activation of PERK and ATF6 [26, 27]. Despite these findings, the role of PRMT1 and its specific substrates in liver fibrosis, particularly in driving HSC activation, has never been explored.

Here, we demonstrate that PRMT1 asymmetrically dimethylated GRP94 at arginine 395. This methylation stabilizes GRP94 protein, contributing to promote the activation of HSCs by ER stress. The PRMT1 inhibitor MS023 inhibits GRP94 asymmetrically dimethylation, thereby suppressing the activation of HSCs and ameliorating CDAHFD-induced or CCl_4_-induced liver fibrosis. These finding provide preclinical evidence supporting further investigation.

## Materials and methods

### Animals

6-week-old male adult C57BL/6J mice were purchased from the Peking University Health Science Animal Center. The mice were housed and maintained under 12 h dark/light cycles, and free access to food and water was provided.

The liver fibrosis model induced by CCl_4_ was implemented according to the previously described method [28]. In summary, mice were administered sterile CCl_4_ (25% v/v dissolved in olive oil, 1 mL/kg) via intraperitoneal injection twice a week for a duration of 3 weeks. In the therapeutic experiments, the duration of model establishment was prolonged to 6 weeks to allow for drug administration. After a 48 h period following the final injection, the mice were euthanized in order to collect serum and liver tissue for subsequent analysis.

The liver fibrosis model induced by A choline-deficient, L-amino acid-defined high-fat diet (CDAHFD) (60 kcal% fat with 0.1% methionine and no added choline, Research Diets) was established based on published literature for 4 weeks [29]. In the therapeutic experiments, the duration of model establishment was prolonged to 8 weeks. After a 48 h period following the final fed, the mice were euthanized in order to collect serum and liver tissue for subsequent analysis.

### Data-independent acquisition (DIA) proteomics

For mouse liver tissues samples, the protein mixtures were extracted, digested, and the peptide mixtures were analyzed with DIA technology. Methodological details are provided in Supplementary material and methods. The data have been deposited to the ProteomeXchange Consortium via the iProX repository with the data set identifier PXD075920.

### Cell culture, transfection, lentivirus infection and CRISPR/Cas9

Human hepatic stellate cells (Procell) were cultured in Dulbecco’s modified Eagle’s medium (DMEM) supplemented with 20% fetal bovine serum (FBS), penicillin (100 U/mL) and streptomycin (100 mg/mL) in 5% CO_2_ at 37 °C. Human hepatic stellate cell line LX2 (Procell) and HEK293T were cultured in DMEM supplemented with 10% fetal FBS at 37 °C in 5% CO_2_. Transfection was performed using the TurboFect transfection reagent (Thermo Scientific, R0533) according to the manufacturer’s protocol. The cells were harvested 48 h after transfection to confirm transfection efficiency. PLVX-IRES-Puro-GRP94, PLVX-IRES-Puro-GRP94^R395A^, PLVX-IRES-Puro-GRP94^R395F^ and PLVX-IRES-Puro-PRMT1 were transfected into LX2 cells to obtain stable transfection cell lines.

The sgRNA targets GRP94 5′-TCTGTTAACTTCGGCTTGGAAGG-3′, targets PRMT1 5′-GATGTCATGTCCTCAGCGTTGGG-3′ and targets USP14 5′- GCTCAGCTGTTTGCGTTGAC-3′ were designed by Zhang Feng’s lab, which was inserted into the CRISPR vector pSpCas9(BB)-2A-Puro (PX459). LX2 cells were transfected with the expression plasmids and screened using puromycin. The knockout efficiency was detected after three weeks. Cell lines were detected every three months. All cells used for experiments were maintained for less than 20 passages.

### Quantitative real-time PCR (qRT-PCR)

Cell RNA was extracted using the RNA simple Total RNA Kit (Tiangen). cDNA was synthesized using the RevertAid First Strand cDNA Synthesis Kit (Thermo Scientific). The mRNA expression level was measured by real-time PCR using SYBR Green qPCR Master Mix (TransGen Biotech). β-actin was used to normalize mRNA expression. Data were analyzed using the relative quantification (ΔΔCt). The primer sequences used are listed in Supplementary Materials and Methods 1.4.

### Western blotting

Cells were lysed in RIPA lysis buffer (Thermo Scientific) containing protease inhibitor cocktail (Sigma), and the BCA protein assay kit (Pierce) was applied to measure the protein concentration. Protein lysates were boiled in 4× SDS loading buffer at 95 °C for 10 min, and separated by SDS-PAGE and were transferred to nitrocellulose membranes (Pall). Infrared fluorescence images were obtained using an Odyssey infrared imaging system (LI-COR Biosciences, Lincoln, NE, USA). The antibodies used are listed in Supplementary Materials and Methods 1.1.

### Immunoprecipitation (IP)

Cells were harvested and lysed in IP lysis buffer (25 mM Tris-HCl (pH 7.4), 150 mM NaCl, 1% Nonidet P-40, 1 mM EDTA, and 5% glycerol) containing 1% protease inhibitor cocktail for 1 h at 4 °C. Then, the protein lysates incubated at 4 °C overnight with antibodies and protein A-Sepharose (GE Healthcare). The beads were washed thrice with IP lysis buffer and boiled in 4× SDS loading buffer at 95 °C for 10 min. The antigen-antibody complexes were subjected to Western blotting with the indicated antibodies.

### GST pull-down

GST and GST-tagged proteins were purified from *Escherichia coli* BL21 (DE3). The purified proteins were then incubated with glutathione-sepharose beads (GE Healthcare) overnight at 4 °C. The next day, samples were incubated with His-tagged proteins for 4 h at 4 °C. After incubation, beads were washed with ice-cold elution buffer three times, The bound antigen-antibody complexes were eluted by boiling in 4× SDS loading buffer, and detected by Western blotting with the indicated antibodies.

### In vitro methylation

The purified GST-PRMT1 and His-GRP94 were incubated with HMT buffer (25 mM Tris-HCl pH 8.8, 25 mM NaCl, 2 μM SAM). The reactions were incubated at 37 °C for 2 h.

### In vivo ubiquitination

Cells were transfected with the relevant plasmids for 36 h. Prior to harvest, the cells were treated with 10 μM MG132 for 6 h. Subsequently, the cells were lysed using immunoprecipitation (IP) lysis buffer. The lysates were then subjected to immunoprecipitation by incubating with the specified antibody and Protein A-Sepharose beads (GE Healthcare) overnight at 4 °C. The following day, the immunoprecipitated complexes were washed three times and analyzed by Western blotting using the designated antibodies.

### Cycloheximide (CHX) chase assay

Cells were added with the cycloheximide (CHX) for 0, 4, 8, 12, and 16 h separately at a final concentration of 100 μg/mL. Then, we collected and lysed cells. The endogenous GRP94 protein levels were detected by Western blotting and normalized by β-actin.

### Cell migration

The migration assay was conducted in transwell chambers containing 8 μm core filters (Corning). 5 × 10^4^ cells were seeded to each upper chamber with 100 μL serum-free medium, and the lower chambers were filled with DMEM supplemented with 10% FBS. Following a 24-h incubation, the invasive cells were fixed with 4% paraformaldehyde (PFA) for 30 min and stained with a 0.1% crystal violet solution. After rinsing with deionized water to remove excess stain, the cells were visualized under a microscope (Leica). Cell counts were performed for at least three independent fields of view using Adobe Photoshop software.

### Immunofluorescence (IF) assay

Cells were cultured in 3.5 cm confocal dishes (Corning) and washed with PBS at room temperature three times. Then, the cells were fixed with 4% PFA for 15 min and permeabilized (with 0.2% Triton X-100 in PBS) for 10 min at room temperature. After washing three times, the cells were blocked with 1% bovine serum albumin (BSA) in PBS for 1 h at room temperature and incubated overnight with primary antibodies at 4 °C. On the second day, the cells were incubated with the secondary antibodies. Nuclei were stained with DAPI at a concentration of 1 μg/ml for 2 min. Images were captured using a fluorescence microscope (Zeiss). Mean fluorescence intensity analyzed using ImageJ and normalized by controls.

### Oil red O staining

Cells were fixed with 4% PFA and then stained at RT for 15 min by adding the oil red O staining solution. Following this, the cells were treated with 60% IPA and stained with drops of hematoxylin solution. After thorough washing, the stained fat droplets within the cells were observed and photographed using a light microscope (Leica). Positively stained areas were analyzed using ImageJ and normalized by controls.

### Enzyme-linked immunosorbent assay (ELISA)

Plasma Hyaluronidase (HAase) (Elabscience) and N-Terminal Procollagen III Propeptide (PNIIIP) (Cloud-Clone) levels were assessed using ELISA kit according to the manufacturer’s protocols.

### Histological analysis and serum biochemistry

Liver tissues had been fixed with 4% paraformaldehyde, embedded in paraffin in order to carry out hematoxylin and eosin (H&E) staining, Masson’s trichrome staining, and Sirius red staining. ImageJ was performed to analyze the stained area of liver tissue sections after staining.

### Statistical analysis

SPSS 17.0 software and GraphPad Prism 9 were used for statistical analysis. Data are expressed as mean ± standard deviation (SD) of at least three independent experiments, where *n* represents biological replicates. Student’s *t* test was used for calculating the differences between two groups. One-way ANOVA was used to evaluate differences among multiple groups. *p* < 0.05 was considered statistically significant. *p < 0.05, ** *p* < 0.01, *** *p* < 0.001; n.s., no significance.

## Results

### GRP94 methylation is elevated in activated HSCs and liver fibrosis

**Fig. 1:**
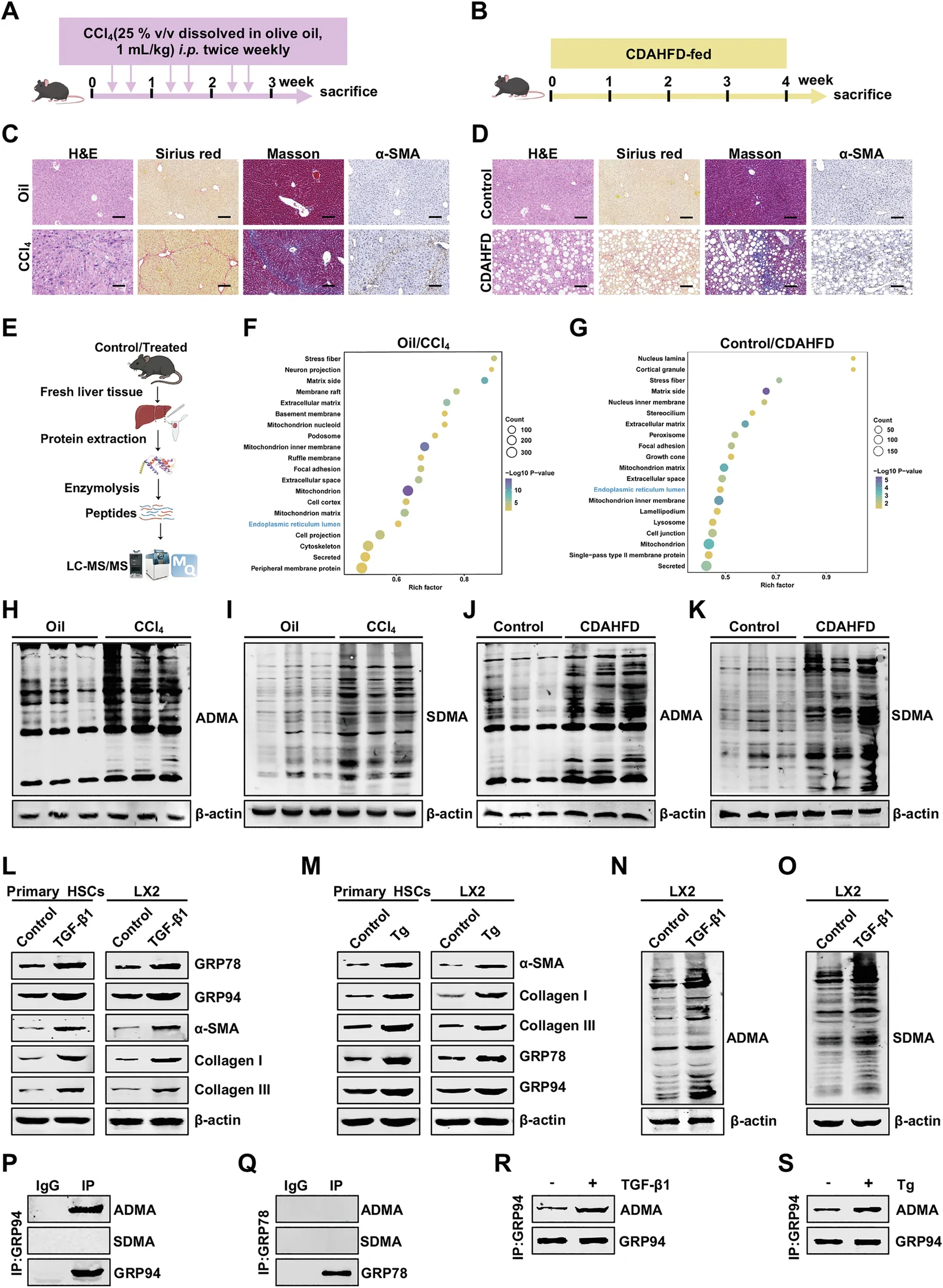
GRP94 methylation is elevated in activated HSCs and liver fibrosis. Schematic illustrations of the experimental design showing the mouse model of **A** CCl_4_-induced and **B** CDAHFD-induced fibrosis (*n* = 5). **C**, **D** Liver tissue sections from CCl_4_-induced or CDAHFD-induced mice were analyzed by hematoxylin and eosin (H&E), Sirius Red, Masson staining as well as immunohistochemistry (IHC) for α-SMA. **E** Schematic overview of the experimental design for proteomic profiling (*n* = 3). **F**, **G** Liver fibrosis in mice is associated with endoplasmic reticulum (ER) stress. Bubble diagram showing the top 20 terms that were significantly enriched in the differentially expressed proteins of the CCl_4_- induced (**F**) or CDAHFD-induced (**G**) liver fibrosis models, based on subcellular localization analysis. **H**–**K** The total protein methylation level was elevated in the livers of mice with fibrosis. The overall levels of ADMA and SDMA in the proteins from liver tissues of mice with (**H**, **I**) CCl_4_- or (**J**, **K**) CDAHFD-induced liver fibrosis was determined by Western blotting. β-actin was used as a loading control. **L** TGF-β1 stimulates the expression of ER stress-related and fibrosis-related proteins in human primary HSCs and LX2 cells. Protein levels of GRP78, GRP94, α-SMA, Collagen I, and Collagen III in human primary HSCs or LX2 cells treated with or without TGF-β1(10 ng/mL) were measured by Western blot. β-actin was used as a loading control. **M** Tg increases the expression of fibrosis-related and ER stress-related proteins in human primary HSCs and LX2 cells. Protein levels of α-SMA, Collagen I, Collagen III, GRP78, and GRP94 in human primary HSCs or LX2 cells treated with or without Tg (1 μmol/L) were measured by Western blot. **N**, **O** TGF-β1 promotes the global methylation modification level in LX2 cells. The overall ADMA (**N**) and SDMA (**O**) level of proteins in LX2 cells were determined by Western blot. **P**, **Q** Endogenous GRP94 undergoes methylation modification, but GRP78 does not. **P** Endogenous GRP94 immunoprecipitates from LX2 cells were immunoblotted with anti-GRP94, anti-ADMA or anti-SDMA antibodies. **Q** Endogenous GRP78 immunoprecipitates from LX2 cells were immunoblotted with anti-GRP78, anti-ADMA, or anti-SDMA antibodies. **R** TGF-β1 promotes the ADMA modification level of GRP94. LX2 cells were treated with or without TGF-β1(10 ng/mL), and endogenous GRP94 immunoprecipitates were immunoblotted with anti-GRP94 or anti-ADMA antibodies. **S** Tg promotes the ADMA level of GRP94. LX2 cells were treated with or without Tg (1 μmol/L), and endogenous GRP94 immunoprecipitates were immunoblotted with anti-GRP94 or anti-ADMA antibodies. Data are presented as the mean ± SD. *p < 0.05, **p < 0.01, ***p < 0.001.

Liver fibrosis is a common pathological phase triggered by diverse etiologies such as viral infection, alcohol, and metabolic stress. Many different liver disease models involve the formation process of liver fibrosis [4]. Here, we established two mouse models of liver fibrosis, namely the CCl_4_-induced model (Fig. 1A) and the CDAHFD-induced model (Fig. 1B). Liver tissues in the model groups exhibited marked fibrosis, inflammation, and severe injury, as shown by hematoxylin and eosin (H&E) staining (Fig. 1C, D). H&E staining also revealed obvious hepatic steatosis in the CDAHFD-induced model (Fig. 1D). Increased collagen deposition and fibrotic regions were observed in both models using Sirius red, Masson, and α-SMA immunostaining (Fig. 1C, D, Supplementary Fig. S1A, B). Compared with controls, mice treated with CCl_4_ or fed a CDAHFD showed elevated serum levels of ALT, AST, CREA, and UA (Supplementary Fig. S1C, D), indicating liver and kidney damage. Serum fibrosis markers HAase and PIIINP were also significantly increased (Supplementary Fig. S1E, F). Quantitative reverse transcription polymerase chain reaction (qRT-PCR) analysis demonstrated a parallel increase in the expression of key genes (*Tgf-β1, Acta2, Col1a1*) associated with the advancement of liver fibrosis (Supplementary Fig. S1G, H). The above results confirm that both models can induce the occurrence of liver fibrosis in mice.

A systematic proteomic analysis was conducted on the liver tissues from two different fibrosis models (Fig. 1E). Subcellular localization (SL) analysis revealed a significant enrichment of differentially expressed proteins in the ‘endoplasmic reticulum lumen’ (Fig. 1F, G). Complementarily, Gene Ontology Molecular Function (GOMF) enrichment analysis identified ‘oxidoreductase activity’ as a significantly enriched term (Supplementary Fig. S1I). This combined evidence strongly suggested that the occurrence of ER stress is accompanied by profound disruption of redox homeostasis in fibrotic liver tissues. Immunoblotting showed elevated ADMA and SDMA levels in fibrotic liver samples compared with normal tissue, indicating an overall heightened protein methylation in both models (Fig. 1H–K). Based on the above findings of persistent ER stress and increased global protein methylation during liver fibrosis, we hypothesized unexplored functional connections among ER stress, protein methylation, and liver fibrosis. Given that HSC activation is the primary mechanism driving liver fibrosis, we conducted the following studies at the cellular level to systematically investigate this hitherto unexplored mechanism.

The LX2 cell line, which is derived from human HSCs and displays key features related to fibrosis, has been extensively studied in the research of liver fibrosis [30]. Consequently, we conducted cell-level verification using LX2 cells and human primary hepatic stellate cells (primary HSCs). Treatment with the HSC activator TGF-β1 (10 ng/ml) increased expression of fibrotic markers (α-SMA, Collagen I, Collagen III) and ER stress-related proteins GRP78 and GRP94 in both primary HSCs and LX2 cells (Fig. 1L). Similarly, treatment with the ER stress inducer thapsigargin (Tg, 1 μmol/L) upregulated both fibrosis related and ER stress-related proteins (Fig. 1M). These results indicate that HSC activation promotes ER stress, and conversely, ER stress induction enhances HSC activation. Under TGF-β1 stimulation, global ADMA and SDMA levels in LX2 cells increased (Fig. 1N, O), suggesting a positive correlation between HSC activation, ER stress, and protein methylation. We speculate that these two processes may not be independent but interact through post-translational methylation modifications of key proteins involved in ER stress. We therefore examined methylation of the ER stress-related proteins GRP78 and GRP94. Immunoprecipitation of endogenous proteins from LX2 cells followed by immunoblotting with ADMA and SDMA antibodies revealed that GRP94, but not GRP78, underwent ADMA modification (Fig. 1P, Q). More importantly, GRP94 methylation levels were significantly increased upon treatment with TGF-β1 (Fig. 1R) or Tg (Supplementary Fig. 1S). Collectively, these results demonstrate that HSC activation is associated with increased global protein methylation, and that GRP94, a master chaperone in ER stress, undergoes methylation that is further enhanced upon HSC activation and ER stress induction.

### GRP94 promotes the activation of HSCs by enhancing ER stress

**Fig. 2:**
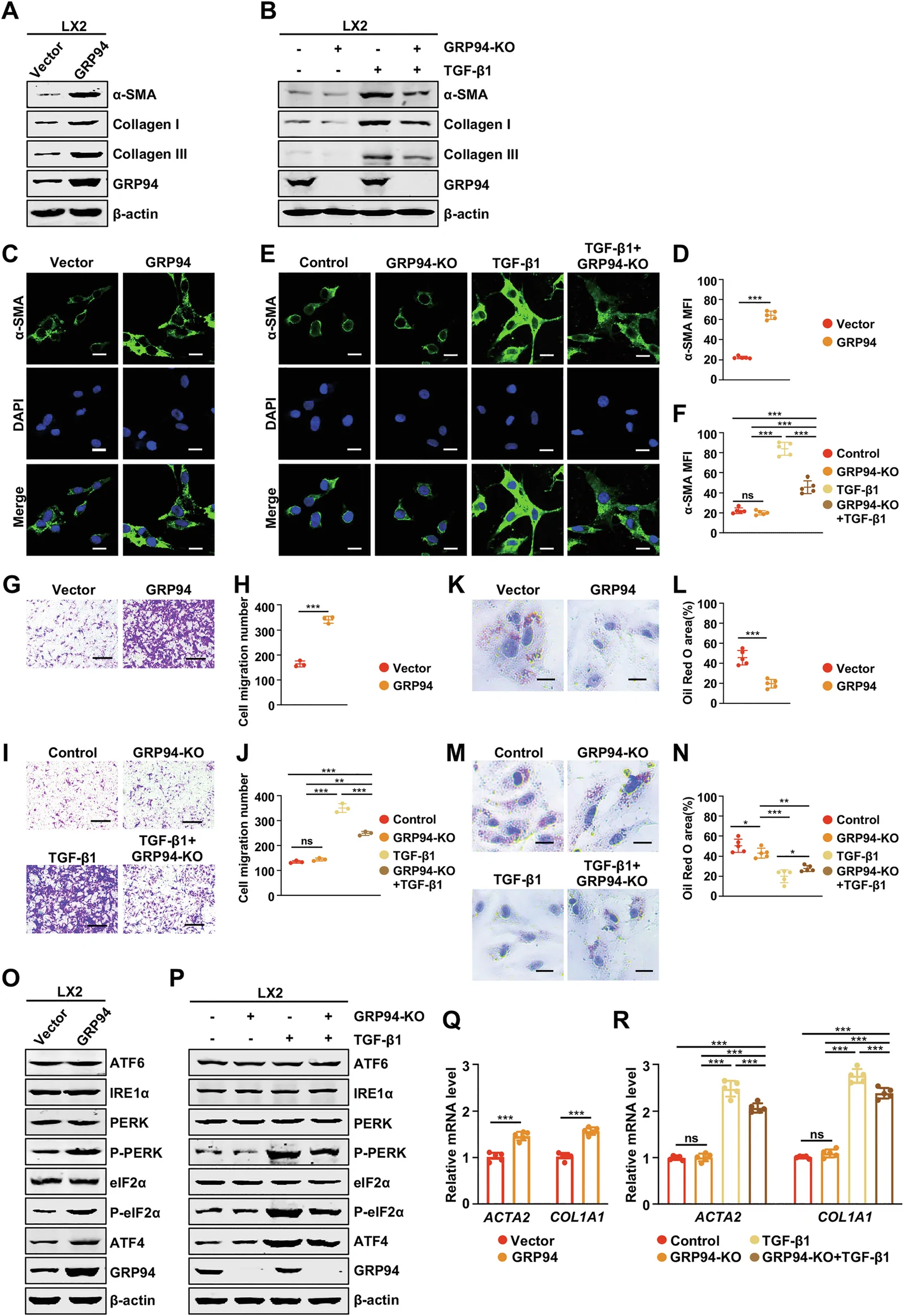
GRP94 promotes the activation of HSCs by ER stress. **A**–**F** GRP94 promotes the expression of activation marker proteins in HSCs. **A** Western blot analysis of α-SMA, Collagen I, and Collagen III protein in LX2 cells with or without GRP94 overexpression. β-actin was used as a loading control. **B** Western blot was performed to determine the protein expression of α-SMA, Collagen I, and Collagen III in wild-type (WT) or GRP94-knockout (KO) LX2 cells treated with or without TGF-β1(10 ng/mL). β-actin was used as a loading control. **C** Representative immunofluorescence (IF) images and **D** quantification of mean fluorescence intensity (MFI) for α-SMA expression in LX2 cells with GRP94 overexpression. α-SMA (green), nucleus (blue). Scale bar, 50 μm. **E** Representative IF images and **F** quantification of MFI for α-SMA expression in WT or GRP94-KO LX2 cells treated with or without TGF-β1(10 ng/mL). α-SMA (green), nucleus (blue). Scale bar, 50 μm. **G**–**I** GRP94 promotes the migration of hepatic stellate cells. **G** Representative images and **H** quantification of migration cells from transwell assays in GRP94-overexpressing LX2 cells. Scale bar, 500 µm. **I** Representative images and **J** quantification migrated cells from transwell assays in WT or GRP94-KO LX2 cells treated with or without TGF-β1(10 ng/mL). Scale bar, 500 µm. **K**–**N** GRP94 inhibits lipid droplet storage in HSCs. **K** Representative images and **L** quantification of Oil Red O staining in GRP94-overexpressing LX2 cells, scale bar, 50 µm. **M** Representative images and **N** quantification of Oil Red O staining in WT or GRP94-KO LX2 cells treated with TGF-β1(10 ng/mL). Scale bar, 50 µm. **O**, **P** GRP94 promotes the expression of ER stress-related proteins. **O** The levels of ER stress-related proteins in LX2 cells with or without GRP94 overexpression were measured by Western blot. β-actin was used as a loading control. **P** Western blot analysis was performed to determine the expression of ER stress-related proteins in WT or GRP94-KO LX2 cells treated with or without TGF-β1(10 ng/mL). β-actin was used as a loading control. **Q** GRP94 upregulates the mRNA levels of hepatic stellate cell activation markers. The mRNA levels of *ACTAC2* and *COL1A1* in LX2 cells with or without GRP94 overexpression were measured by qRT-PCR. **R** qRT-PCR analysis of *ACTA2* and *COL1A1* mRNA levels in WT or GRP94-KO LX2 cells treated with or without TGF-β1 (10 ng/mL). β-actin was used as a control. Data are presented. as the mean ± SD. *p < 0.05, **p < 0.01, ***p < 0.001.

Given that GRP94 expression is elevated in active primary HSCs and LX2 cells, we hypothesized that the high expression of GRP94 could positively promote cell activation. Thus, we constructed cell lines with stable GRP94 overexpression or knockout (KO). Activated HSCs are characterized by increased proliferation and migration, upregulated expression of fibrosis-related proteins (α-SMA, Collagen I, Collagen III), and reduced lipid droplet storage [28, 31]. We conducted experimental verification of the aforementioned activated phenotypes. Western blotting showed that GRP94 overexpression increased the expression of α-SMA, Collagen I, and Collagen III (Fig. 2A). In the quiescent state, the GRP94 KO has no significant effect on the LX2 cells. To explore whether GRP94 knockout inhibits HSC activation, we added the activation inducer TGF-β1 as a positive control. GRP94 KO suppressed the expression of these fibrosis-related proteins induced by TGF-β1 (Fig. 2B). Immunofluorescence (IF) detection for α-SMA yielded consistent results (Fig. 2C–F). Cell migration experiment showed that GRP94 promotes the migration of LX2 (Fig. 2G, H), conversely, GRP94 KO suppressed the pro-migration effect of TGF-β1 (Fig. 2I, J). Subsequently, Oil Red O staining was conducted to visualize the lipid droplets in LX2 cells. The results indicated that GRP94 markedly attenuated lipid droplet storage (Fig. 2K, L), and importantly, GRP94 KO reversed the inhibitory effect of TGF-β1 on this process (Fig. 2M, N). In addition, we examined the protein levels of three key receptors of ER stress (ATF6, IRE1α and P-PERK) to explore the specific role of GRP94 in ER stress in HSC. GRP94 overexpression increased levels of P-PERK and its downstream targets P-eIF2α and ATF4, whereas GRP94 KO inhibited TGF-β1-induced activation of this pathway (Fig. 2O, P). Neither overexpression nor KO of GRP94 affected the protein levels of ATF6 and IRE1α. These results demonstrate that GRP94 can activate the PERK-eIF2α-ATF4 pathway in ER stress. Previous studies have shown that the transcription factor ATF4 can transcriptionally activate genes *ACTA2* and *COL1A1* related to fibrosis [32]. Therefore, we assumed that GRP94 could enhance this effect. qRT-PCR confirmed that GRP94 promoted ATF4-mediated transcriptional activation of *ACTA2* and *COL1A1* (Fig. 2Q). GRP94 KO suppressed this induction by TGF-β1 (Fig. 2R). The data indicate that GRP94 promotes the HSC activation by stimulating the PERK-eIF2α-ATF4 pathway.

### GRP94 is methylated by PRMT1 at arginine 395

**Fig. 3:**
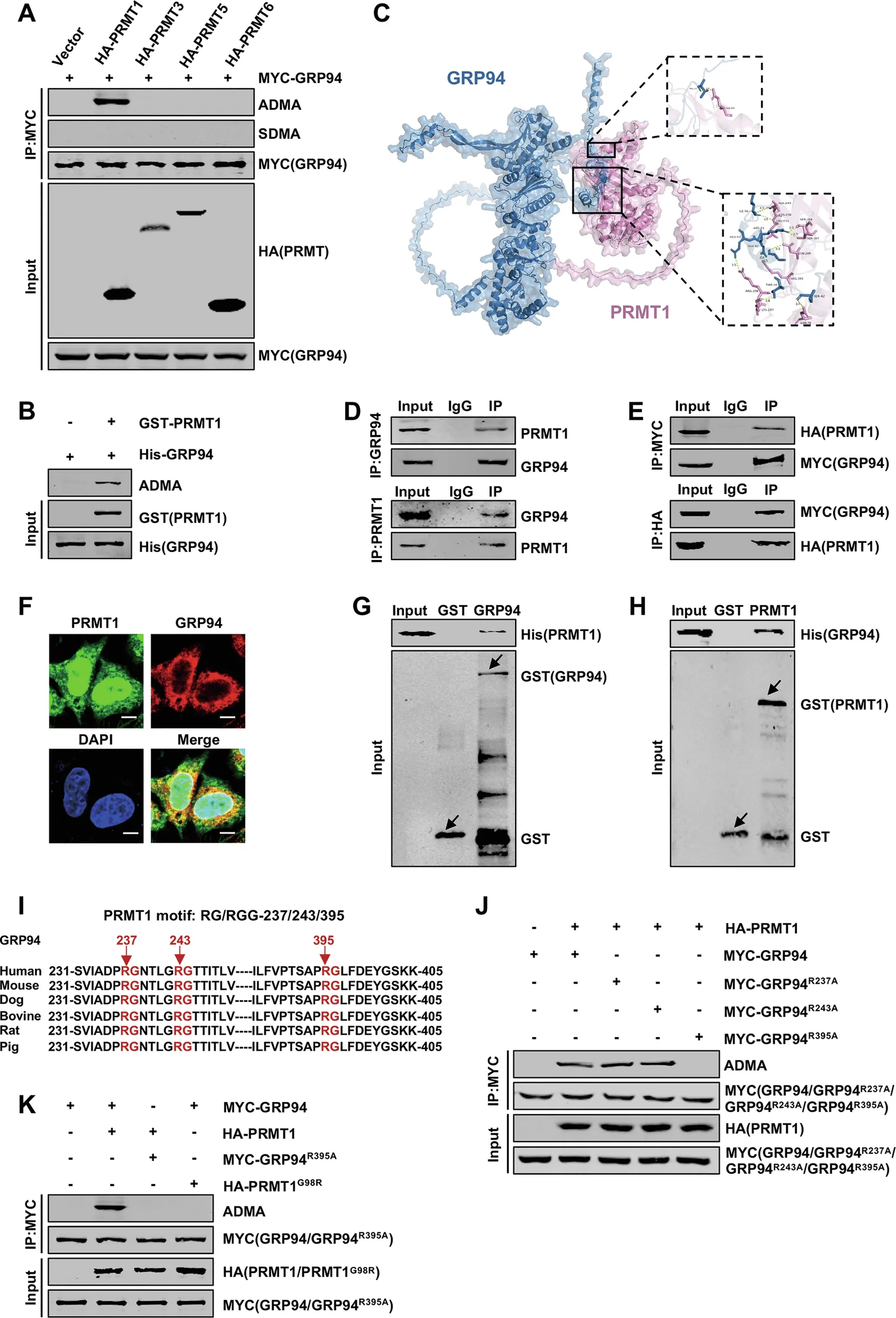
GRP94 is asymmetrically dimethylated by PRMT1 at arginine 395. **A** GRP94 is asymmetrically dimethylated by PRMT1 in vivo. HEK293T cells were co-transfected with MYC-GRP94 and HA-PRMT1/PRMT3/PRMT5/PRMT6. Total cell lysates were immunoprecipitated with an anti-MYC antibody and detected by Western blotting. **B** GRP94 is asymmetrically methylated by PRMT1 in vitro. Purified His-GRP94 was incubated with GST or GST-PRMT1 in histone methyltransferase (HMT) buffer for 2 h at 37 °C, following by Western blotting with anti-His, anti-GST, and anti-ADMA antibodies. **C**–**F** GRP94 interacts with PRMT1 in vivo. **C** AlphaFold prediction of the complex structure of GRP94 and PRMT1. **D** Interaction between endogenous GRP94 and PRMT1 in LX2 cells. LX2 cells lysates were immunoprecipitated with anti-GRP94 or anti-PRMT1 antibodies and immunoblotted with indicated antibodies. **E** The interaction between exogenous GRP94 and PRMT1 in HEK293T cells. HEK293T cells were co-transfected with MYC-GRP94 and HA-PRMT1. Lysates were subjected to co-immunoprecipitation (Co-IP) using anti-MYC or anti-HA antibodies, followed by Western blotting. **F** IF images demonstrating the colocalization of GRP94 and PRMT1 in the cytoplasm. LX2 cells were stained with anti-GRP94 antibody (red) and anti-PRMT1 antibody (green). nucleus (blue). Scale bar, 50 μm. **G**, **H** GRP94 interacts with PRMT1 in vitro. **G** GST pull-down assay using His-PRMT1 incubated with either GST or GST-GRP94, followed by Western blotting with an anti-His antibody. **H** GST pull-down assay was performed with His-GRP94 and incubated with either GST or GST-PRMT1, followed by Western blotting with an anti-His antibody. **I**–**K** PRMT1 asymmetrically methylates GRP94 at arginine 395. **I** Schematic representation of the GRP94 methylation site mutation strategy. **J** HEK293T cells were co-transfected with MYC-GRP94/GRP94^R237A^/GRP94^R243A^/GRP94^R395A^ and HA-PRMT1(or empty vector). Lysates were immunoprecipitated with an anti-MYC antibody and immunoblotted as indicated. **K** HEK293T cells were co-transfected with MYC-GRP94/GRP94^R395A^ and HA-PRMT1/HA-PRMT1^G98R^. Lysates were immunoprecipitated with an anti-MYC antibody and immunoblotted as indicated.

The results shown in Fig. 1P indicate that endogenous GRP94 undergoes ADMA modification in LX2. However, the enzyme that catalyzes this methylation and the specific methylation sites have not yet been investigated. To identify the specific methylating enzyme, we individually co-transfected MYC-GRP94 with predominant methylating enzyme (HA-PRMT1, 3, 5 and 6) in HEK293T cells. Immunoprecipitation (IP) analysis indicated that GRP94 was asymmetrically dimethylated specifically by PRMT1 in vivo (Fig. 3A). An in vitro methylation assay further confirmed that recombinant His-GRP94 was asymmetrically dimethylated upon addition of GST-PRMT1 (Fig. 3B). These data confirm that PRMT1 methylates GRP94 both in vivo and in vitro. Next, we investigated whether GRP94 combines with PRMT1, we employed AlphaFold to predict their complex structure. The Fig. 3C shows the top-ranked binding interface. Then, we confirmed the endogenous binding of GRP94 to PRMT1 through Co-immunoprecipitation (Co-IP) experiments (Fig. 3D). In HEK293T cells, we ectopically expressed MYC-GRP94 and HA-PRMT1 and performed Co-IP experiment. The results showed that exogenous GRP94 could bind to PRMT1 in vivo (Fig. 3E). Immunofluorescence staining further confirmed co-localization of GRP94 and PRMT1 in the cytoplasm (Fig. 3F). Finally, we performed a GST pull-down assay, which demonstrated the direct interaction between GRP94 and PRMT1 in vitro (Fig. 3G, H). It has been reported that the modification site of PRMT1 is arginine in RG or RGG [33, 34]. Analysis of GRP94 coding sequence revealed three potential methylation sites: R237, R243, and R395 (Fig. 3I). We generated site-specific mutants (Arg/Ala) (GRP94^R237A^, GRP94^R243A^, GRP94^R395A^) and a catalytically inactive PRMT1 mutant (PRMT1^G98R^) [33, 34]. Then, HA-PRMT1 and MYC-GRP94, MYC-GRP94^R237A^, MYC-GRP94^R243A^ or MYC-GRP94^R395A^ were co-transfected respectively in HEK293T cells to test whether GRP94 could be methylated by PRMT1. As shown in Fig. 3J, Co-IP experiment confirmed that GRP94^R395A^ mutant could not be methylated by PRMT1. To further confirm that PRMT1 methylates GRP94 at R395 site, we co-transfected MYC-GRP94 or MYC-GRP94^R395A^ together with HA-PRMT1 or PRMT1^G98R^ into HEK293T cells. Methylation occurred only with wild-type GRP94 and PRMT1 transfected (Fig. 3K), indicating that PRMT1 specifically targets R395 site of GRP94. To further confirm the specific methylated arginine residue in GRP94, we performed liquid chromatography tandem mass spectrometry (LC-MS/MS)-based proteomic analysis of endogenous GRP94 in HEK293T cells. We detected methylation at R395 of endogenous GRP94 in cell extracts (Supplementary Fig. S2A and Supplementary Table 1).

### GRP94 methylation by PRMT1 increases GRP94 stability by inhibiting its ubiquitination

**Fig. 4:**
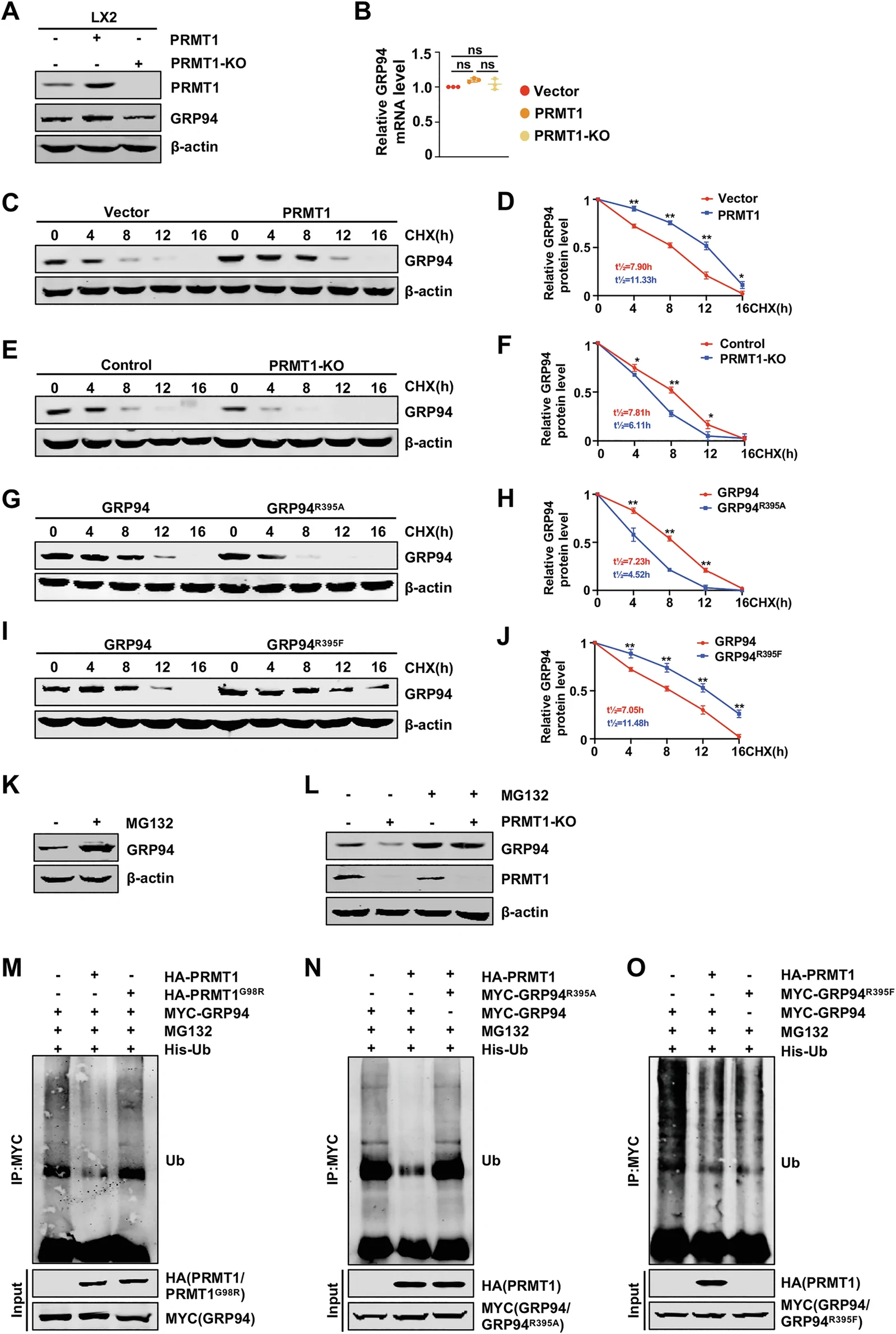
GRP94 methylation by PRMT1 increases GRP94 stability by inhibiting its ubiquitination. **A**, **B** PRMT1 upregulates GRP94 expression. **A** Western blot analysis of GRP94 protein levels in WT, PRMT1 overexpression, and PRMT1-KO LX2 cells. **B** Quantitative RT-PCR analysis of endogenous GRP94 mRNA levels in WT, PRMT1-OE, and PRMT1-KO LX2 cells. **C**–**F** PRMT1 increases GRP94 protein stability. **C** LX2 cells transfected with empty vector or PRMT1 were treated with cycloheximide (CHX, 100 μg/mL) for 0, 4, 8, 12, or 16 h (*n* = 3). GRP94 protein levels were detected by Western blotting. **D** Quantification of GRP94 protein half-life from the experiments shown in (**C**). Data are presented as the mean ± SD (*n* = 3). **E** Western blot analysis of endogenous GRP94 expression in control or PRMT1-KO LX2 cells treated with CHX for the indicated times. **F** Quantification of GRP94 protein half-life from the experiments shown in (**E**). Data are presented as the mean ± SD (*n* = 3). **G**–**J** The methylation of GRP94 increases its protein stability. **G** LX2 cells transfected with MYC-GRP94 or MYC-GRP94^R395A^ were treated with cycloheximide (CHX, 100 μg/mL) for 0, 4, 8, 12, or 16 h. GRP94 protein levels were detected by Western blotting. **J** LX2 cells transfected with MYC-GRP94 or MYC-GRP94^R395F^ were treated with cycloheximide (CHX, 100 μg/mL) for the indicated times (*n* = 3). GRP94 protein levels were detected by Western blotting. **H**, **I** Quantification of GRP94 protein half-life from the experiments shown in (**G**) and (**J**), respectively. Data are presented as the mean ± SD (*n* = 3). **K**, **L** PRMT1 stabilizes GRP94 by inhibiting proteasomal degradation. **K** LX2 cells were treated with or without the proteasome inhibitor MG132 for 12 h, and GRP94 protein levels were measured by Western blotting. **L** Control or PRMT1-KO LX2 cells were treated with or without MG132 for 12 h, and GRP94 protein levels were detected by Western blotting. **M** PRMT1-mediated methylation of GRP94 at arginine 395 inhibits its ubiquitination. HEK293T cells were co-transfected with His-ubiquitin, MYC-GRP94, and either HA-PRMT1 or HA-PRMT1^G98R^. Cells were incubated with MG132 for 12 h, then immunoprecipitated with an anti-MYC antibody. Ubiquitination of GRP94 was detected by Western blotting with an anti-ubiquitin antibody. **N**, **O** Asymmetrically methylation of GRP94 is required for the inhibition of its ubiquitination. **N** HEK293T cells were co-transfected with His-ubiquitin, HA-PRMT1(or empty vector), and either MYC-GRP94 or MYC-GRP94^R395A^. Following MG132 treatment for 12 h, lysates were immunoprecipitated with anti-MYC antibody and immunoblotted for ubiquitin. **O** HEK293T cells were co-transfected His-ubiquitin, HA-PRMT1(or empty vector), and either MYC-GRP94 or MYC-GRP94^R395F^, incubated with MG132 for 12 h and then immunoprecipitated with anti-MYC antibody. The GRP94 ubiquitination was measured by Western blotting. Data are presented as the mean ± SD. *p < 0.05, **p < 0.01, ***p < 0.001.

Notably, increasing evidence has illuminated that the non-histone arginine methylation catalyzed by PRMT1 has implication on their stability [21, 35]. Meanwhile, we found that the expression levels of PRMT1 and GRP94 in liver tissues were significantly upregulated when liver fibrosis occurred (Supplementary Fig. S1J, K). To clarify whether the PRMT1-mediated methylation of GRP94 can regulate its expression, we investigated the effects of PRMT1 overexpression or knockout on the protein and mRNA levels of GRP94 using Western blotting and qRT-PCR. Overexpression of PRMT1 increased the protein level of GRP94 in LX2 cells, while knockout of PRMT1 decreased its protein level (Fig. 4A). However, there was no significant change in mRNA levels among different groups (Fig. 4B). These results suggest that PRMT1 enhances GRP94 protein expression at the post-transcriptional level. To investigate how PRMT1 regulates GRP94, we conducted protein half-life experiments to assess the stability of GRP94. PRMT1 overexpression clearly prolonged the half-life of GRP94 from 7.90 to 11.33 h (Fig. 4C, D), whereas knockout of PRMT1 significantly shortened the half-life from 7.81 to 6.11 h (Fig. 4E, F). To further determine whether the enhanced stability of GRP94 is attributed to methylation of R395 site, we constructed a methylation-deficient (Arg/Ala) mutant GRP94^R395A^. In addition, we also constructed a positive (Arg/Phe) mutation, GRP94^R395F^, that simulates constitutive arginine methylation by replacing arginine with phenylalanine, which has similar sized hydrocarbon chains and carries a large hydrophobic moiety. According to the previous report, the hydrophobicity induced by Phe mutation is specific to the methylatable arginine residues, and can simulate the methylation of arginine [33, 36]. Next, wild-type GRP94, GRP94^R395A^ or GRP94^R395F^ were expressed in LX2 cells. It is notable that in the cells treated with chloramphenicol (CHX), the level of the GRP94^R395A^ mutant protein decayed at a faster rate than that of the wild-type GRP94 (Fig. 4G, H). Compared with the wild-type, the GRP94^R395F^ mutant extended the half-life of HBP1 from 7.05 to 11.48 h (Fig. 4I, J). Overall, the methylation of GRP94 at the R395 site by PRMT1 can enhance GRP94 stability.

We assumed that the methylation of GRP94 might enhance the stability of GRP94 by inhibiting its ubiquitination. We incubated LX2 cells with a proteasome degradation inhibitor, MG132, and evaluated the protein level of GRP94 using Western blotting. The results showed that MG132 treatment significantly increased the protein level of GRP94 (Fig. 4K), and this accumulation was not affected by PRMT1 knockout (Fig. 4L), indicating that PRMT1-mediated methylation increases the stability of GRP94 in a proteasome-dependent manner. To determine whether GRP94 methylation inhibits its ubiquitination and degradation, we used HEK293T cells expressing MYC-GRP94 with HA-PRMT1 or HA-PRMT1^G98R^, then detected GRP94 ubiquitination using an anti-multiubiquitin antibody. The ubiquitination level of GRP94 in the HA-PRMT1 transfection group was significantly lower than that in the control group, while this effect was not observed in the HA-PRMT1^G98R^ transfection group (Fig. 4M), indicating that PRMT1 inhibits the ubiquitination process of GRP94. Furthermore, we used methylation-deficient mutant GRP94^R395A^ and simulated methylation mutant GRP94^R395F^ to confirm the result. As shown in Fig. 4N, O, GRP94^R395A^ reversed the inhibitory ubiquitination effect of PRMT1 (Fig. 4N), while GRP94^R395F^ significantly reduced the ubiquitination level even without PRMT1 (Fig. 4O), suggesting that GRP94 methylation by PRMT1 at the R395 site suppresses its ubiquitination.

**Fig. 5:**
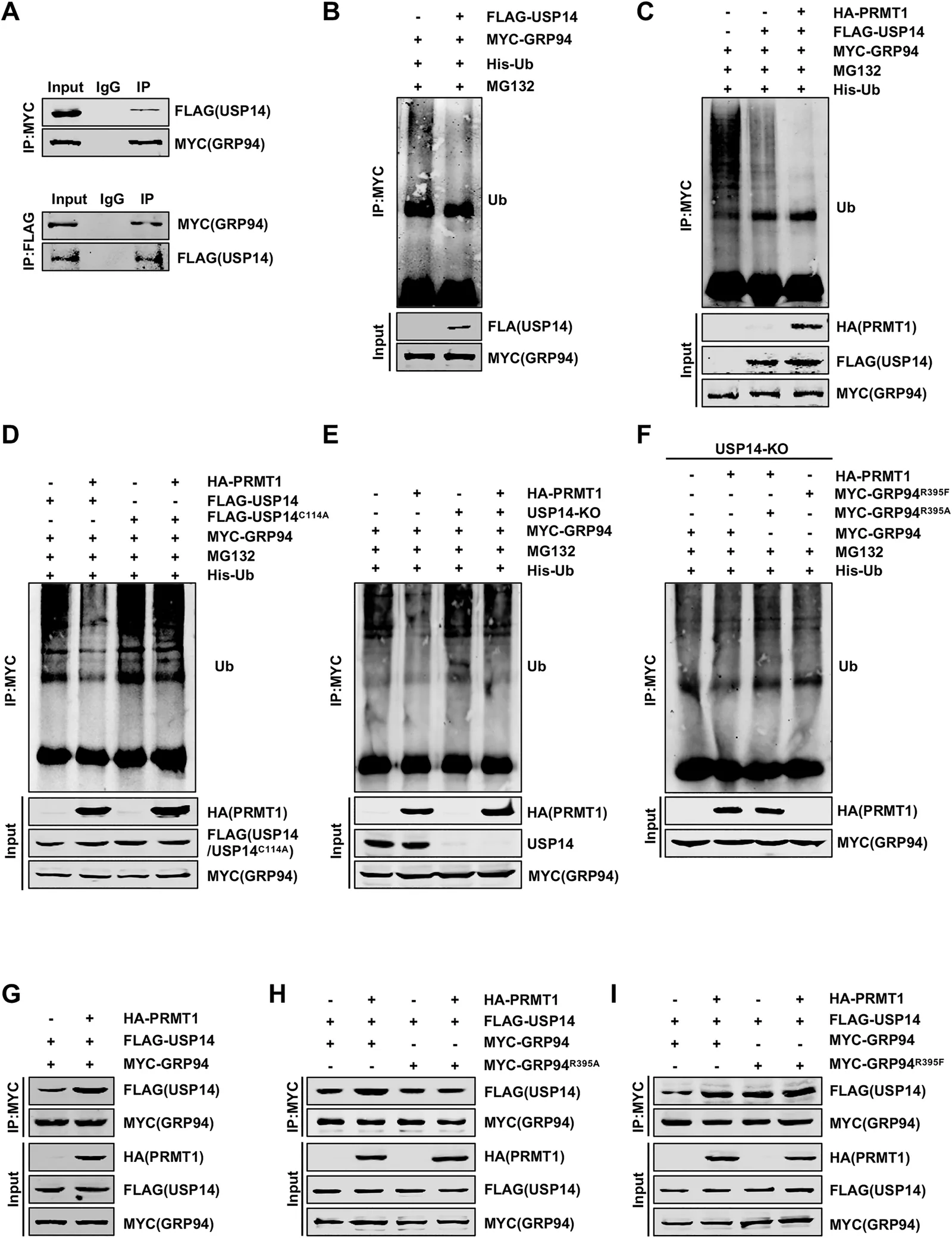
GRP94 methylation by PRMT1 reduces its ubiquitination via promoting USP14 binding. **A**, **B** USP14 is the deubiquitinating enzyme of GRP94. **A** The interaction between exogenous GRP94 and USP14 in HEK293T cells. HEK293T cells were co-transfected with MYC-GRP94 and FLAG-USP14. Lysates were subjected to co-immunoprecipitation (Co-IP) with anti-MYC/HA antibodies, followed by western blotting with the indicated antibodies. **B** HEK293T cells were co-transfected His-ubiquitin, MYC-GRP94, HA-PRMT1 and FLAG-USP14(or empty vector). Lysates were immunoprecipitated with anti-MYC antibody and immunoblotted for ubiquitin. **C**–**F** PRMT1 promotes USP14-mediated deubiquitination of GRP94. **C** HEK293T cells were co-transfected with His-ubiquitin, MYC-GRP94, FLAG-USP14(or empty vector), and HA-PRMT1(or empty vector). Following MG132 treatment, lysates were immunoprecipitated with anti-MYC antibody and immunoblotted for ubiquitin. **D** HEK293T cells were co-transfected with His-ubiquitin, MYC-GRP94, FLAG-USP14/USP14^C114A^), and HA-PRMT1(or empty vector). Following MG132 treatment, lysates were immunoprecipitated with anti-MYC antibody and immunoblotted for ubiquitin. **E** HEK293T wild type cells or USP14-KO cells were co-transfected with His-ubiquitin, MYC-GRP94, and HA-PRMT1(or empty vector). Following MG132 treatment, lysates were immunoprecipitated with anti-MYC antibody and immunoblotted for ubiquitin. **F** HEK293T USP14-KO cells were co-transfected with His-ubiquitin, MYC-GRP94/GRP94^R395A^/GRP94^R395F^), and HA-PRMT1(or empty vector). Following MG132 treatment, lysates were immunoprecipitated with anti-MYC antibody and immunoblotted for ubiquitin. **G**–**I** PRMT1-mediated methylation of GRP94 enhances its binding with USP14. **G** HEK293T cells were co-transfected with MYC-GRP94, FLAG-USP14, and HA-PRMT1(or empty vector). Lysates were subjected to Co-IP with an anti-MYC antibody, followed by Western blotting with anti-FLAG and anti-MYC antibodies. **H** HEK293T cells were co-transfected with MYC-GRP94/GRP95^R395A^, FLAG-USP14, and HA-PRMT1(or empty vector). Lysates were subjected to Co-IP with an anti-MYC antibody, followed by Western blotting with anti-FLAG and anti-MYC antibodies. **I** HEK293T cells were co-transfected with MYC-GRP94/GRP95^R395F^, FLAG-USP14, and HA-PRMT1(or empty vector). Lysates were subjected to Co-IP with an anti-MYC antibody, followed by Western blotting with anti-FLAG and anti-MYC antibodies.

GRP94 methylation by PRMT1 reduces its ubiquitination via promoting USP14 binding USP14 has been identified as a deubiquitinating enzyme for GRP94 [37]. USP14 bound to GRP94 (Fig. 5A), reducing its ubiquitination level (Fig. 5B). Notably, PRMT1 promoted the deubiquitination of USP14 (Fig. 5C). We constructed a previously reported USP14 catalytically inactive mutant USP14^C114A^ [37] and found that PRMT1-mediated deubiquitination of GRP94 depends on the deubiquitinase activity of USP14 (Fig. 5D). A USP14-KO cell line was generated for validation purposes. Ubiquitination analysis demonstrated that in USP14‑KO cells, GRP94 ubiquitination was unaffected by either PRMT1 or GRP94 methylation (Fig. 5E, F), indicated that the effect of PRMT1-mediated methylation of GRP94 on its ubiquitination is dependent on the USP14. The Co-IP experiment further confirmed that PRMT1 promoted the binding of GRP94 to USP14 (Fig. 5G). As shown in Fig. 5H, I, GRP94^R395A^ reversed the promoting binding effect of PRMT1 on GRP94 and USP14 (Fig. 5H), while GRP94^R395F^ was able to promote the binding of GRP94 and USP14 even in the absence of PRMT1 (Fig. 5I). Altogether, our results demonstrate that PRMT1-mediated methylation of GRP94 at arginine 395 promotes USP14-dependent deubiquitination, thereby maintaining GRP94 protein stability.

### GRP94 methylation by PRMT1 promotes the activation of HSCs

**Fig. 6:**
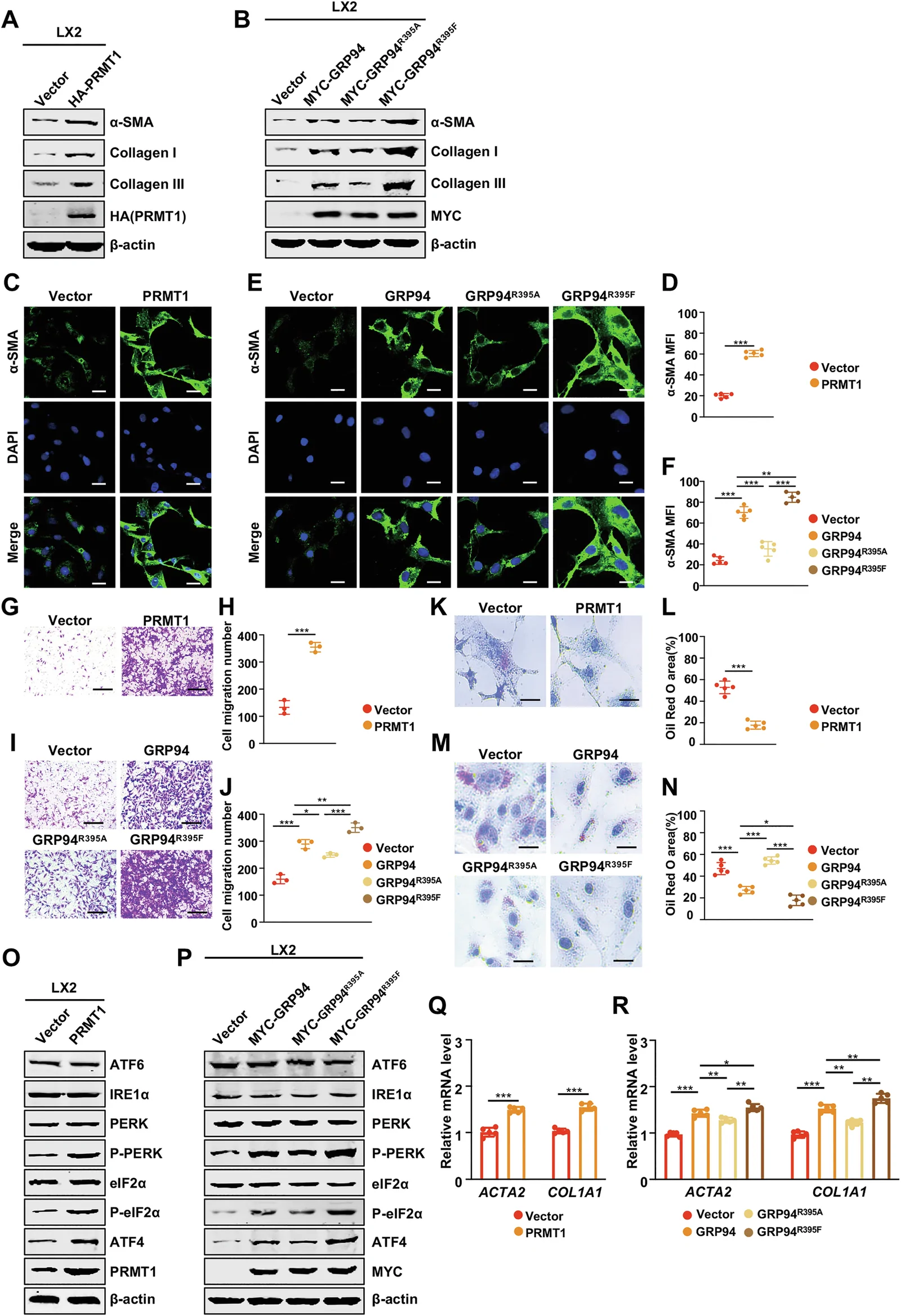
GRP94 methylation by PRMT1 promotes the activation of HSCs. **A**–**F** PRMT1-mediated methylation of GRP94 promotes the expression of activation marker proteins in HSCs. **A** Western blot analysis of α-SMA, Collagen I, and Collagen III protein levels in LX2 cells transfected with or without PRMT1. β-actin was used as a loading control. **B** Western blot analysis of α-SMA, Collagen I, and Collagen III protein levels in LX2 cells transfected with MYC-GRP94/GRP94^R395A^/GRP94^R395F^. β-actin was used as a loading control. **C** Representative IF images and bar blot (**D**) for Mean fluorescence intensity (MFI) for α-SMA expression in LX2 cells with or without PRMT1 transfected. α-SMA (green), nucleus (blue). Scale bar, 50 μm. **E** Representative IF images and **F** quantification of MFI for α-SMA expression in LX2 cells with MYC-GRP94/GRP94^R395A^/GRP94^R395F^ transfected. α-SMA (green), nucleus (blue). Scale bar, 50 μm. **G**–**J** PRMT1-mediated asymmetric methylation of GRP94 promotes HSCs migration. **G** Representative images and cell migration number bar plot **H** of migration cells from transwell assays in LX2 cells transfected with or without PRMT1, scale bar, 500 µm. **I** Representative images and cell migration number bar plot **J** of migration cells from transwell assays in LX2 cells transfected with MYC-GRP94/GRP94^R395A^/GRP94^R395F^. Scale bar, 500 µm. **K**–**N** PRMT1-mediated asymmetric methylation of GRP94 inhibits lipid droplet storage in HSCs. **K** Representative images and quantification of Oil Red O staining area (**L**) in LX2 cells with or without PRMT1overexpression. Scale bar, 50 µm. Representative images (**M**) and quantification of Oil Red O staining area (**N**) in LX2 cells transfected with MYC-GRP94/GRP94^R395A^/GRP94^R395F^. Scale bar, 50 µm. **O**, **P** PRMT1-mediated asymmetric methylation of GRP94 promotes the expression of ER stress-related proteins **O** Western blot was performed to determine the expression of ER stress-related proteins in LX2 cells with or without PRMT1 overexpression. β-actin was used as a loading control. **P** Western blot analysis of ER stress-related proteins in LX2 cells transfected with MYC-GRP94/GRP94^R395A^/GRP94^R395F^. β-actin was used as a loading control. **Q**, **R** PRMT1-mediated asymmetric methylation of GRP94 upregulates mRNA levels of HSC activation markers. **Q** qRT-PCR analysis of *ACTA2* and *COL1A1* mRNA levels in LX2 cells transfected with or without PRMT1. β-actin was used as an internal control **R** qRT-PCR analysis of *ACTA2* and *COL1A1* mRNA levels in LX2 cells transfected with MYC-GRP94/GRP94^R395A^/GRP94^R395F^, β-actin was used as a control. Data are presented as the mean ± SD. *p < 0.05, **p < 0.01, ***p < 0.001.

Given that GRP94 can promote the activation of HSCs, does the methylation modification of GRP94 by PRMT1 affect its pro-activation function? We stably overexpressed PRMT1 in LX2 cells, and the results of Western blotting and IF assays indicated that PRMT1 could promote the expression of fibrosis-related proteins (α-SMA, Collagen I, Collagen III) (Fig. 6A, C, D). Therefore, we speculate that the PRMT1-mediated methylation of GRP94 can enhance its pro-activation effect on HSCs. We conducted Western blotting and IF assays to measure the expression levels of fibrosis-related proteins in LX2 cells that stably expressed MYC-GRP94, MYC-GRP94^R395A^, MYC-GRP94^R395F^, and the control group. As shown in Fig. 6B, E, F, wild-type GRP94 significantly increased the expression of fibrosis-related proteins compared to the control group. This effect was partially abrogated by the methylation-deficient mutant GRP94^R395A^ and, conversely, was further augmented by the simulated methylation mutant GRP94^R395F^. This indicates that the methylation of GRP94 can promote its role in HSC activation. To further verify the credibility of this result, we conducted additional tests on the activation phenotype of HSCs. The cell migration assays showed that PRMT1 promotes cell migration (Fig. 6G, H). The LX2 cells in the GRP94 group were significantly migrated more than those in the control group. The GRP94^R395A^ group had a reduced cell migration compared to the GRP94 group, while the GRP94^R395F^ group had a significantly increased cell migration compared to the GRP94 group (Fig. 6I, J). We also conducted Oil Red O staining on LX2 cells, and the results showed that PRMT1 reduced intracellular lipid droplets (Fig. 6K, L). Lipid droplet accumulation was significantly reduced in the GRP94 group compared to the control. The GRP94^R395A^ group had more lipid droplets storage than the GRP94 group, whereas the GRP94^R395F^ group had fewer lipid droplets storage than the GRP94 group (Fig. 6M, N). We also verified the impact on the ER stress pathway. Consistent with previous studies, overexpression of PRMT1 activates the PERK-eIF2α-ATF4 pathway (Fig. 6O). The pathway was activated by GRP94 relative to the control, while the GRP94^R395A^ mutant partially suppressed this effect, and further enhanced by the GRP94^R395F^ mutant (Fig. 6P). As for the mRNA levels of *ACTC2* and *COL1A1*, PRMT1 significantly increased them (Fig. 6Q), the GRP94 group also raised the mRNA levels, while GRP94^R395A^ relatively inhibited the mRNA levels, and GRP94^R395F^ group showed a more pronounced increase in mRNA levels compared with the GRP94 group (Fig. 6R). The results indicate PRMT1 can activate ER stress. The simulated continuous methylation mutant of GRP94^R395F^ is able to promote the activation effect on the HSCs more effectively than wild-type GRP94, demonstrating that the PRMT1-mediated methylation of GRP94 can promote the activation of ER stress. Overall, our data demonstrate that GRP94 methylation by PRMT1 promotes the HSC activation through ER stress.

### The PRMT1 inhibitor MS023 suppresses HSCs activation

**Fig. 7:**
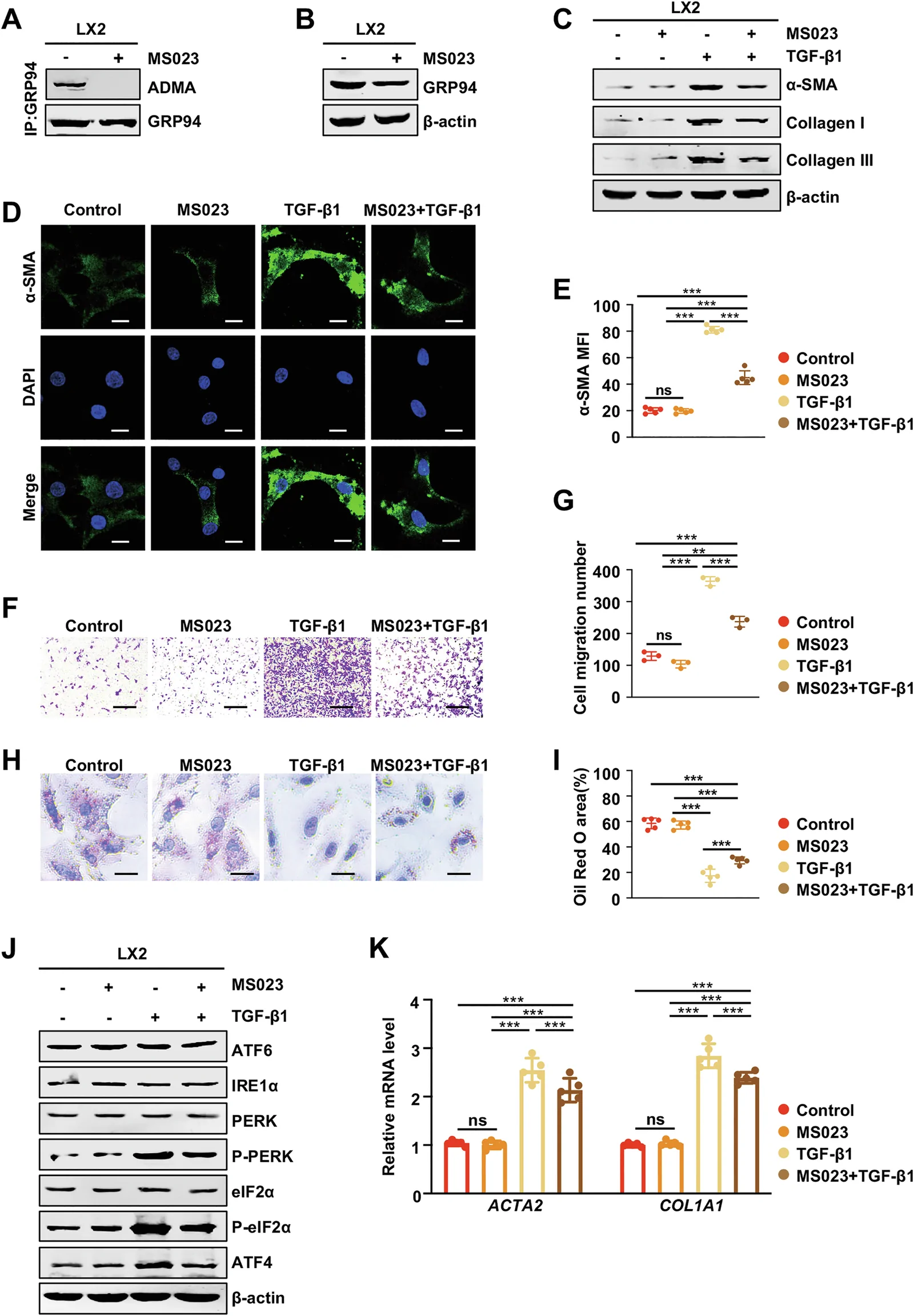
The PRMT1 inhibitor MS023 suppresses HSCs activation. **A** MS023 reduces the ADMA modification level of GRP94. The LX2 cells were treated with or without MS023 (10 μM), respectively, and then the endogenous GRP94 immunoprecipitates were detected by Western blotting using anti-GRP94 or anti-ADMA antibodies. **B** MS023 reduces the protein level of GRP94. Western blot analysis of GRP94 protein levels in LX2 cells treated with or without MS023(10 μM). β-actin was used as a loading control. **C**–**E** MS023 suppresses the expression of activation marker proteins in HSCs. **C** Western blot analysis of α-SMA, Collagen I, and Collagen III protein levels in LX2 cells treated with or without MS023 (10 μM) and TGF-β1 (10 ng/mL). β-actin was used as a loading control. **D** Representative IF images and **E** quantification of MFI for α-SMA expression in LX2 cells treated with or without MS023(10 μM) and TGF-β1(10 ng/mL). α-SMA (green), nucleus (blue). Scale bar, 50 μm. **F**, **G** MS023 inhibits HSC migration. **F** Representative images and **G** quantification of migrated cells from transwell assays in LX2 cells treated with or without MS023(10 μM) and TGF-β1(10 ng/mL), scale bar, 500 µm. **H**, **I** MS023 promotes lipid droplet storage in HSCs. **H** Representative images and **I** quantification of Oil Red O staining area in LX2 cells with or without MS023(10 μM) and TGF-β1(10 ng/mL), scale bar, 50 µm. **J** MS023 reduces the expression of ER stress-related proteins. Western blot analysis of ER stress-related protein expression in LX2 cells treated with or without MS023(10 μM) and TGF-β1(10 ng/mL). β-actin was used as a loading control. **K** MS023 downregulates the mRNA levels of HSC activation markers. The mRNA levels of *ACTAC2* and *COL1A1* in LX2 cells with or without MS023 (10 μM) and TGF-β1(10 ng/mL) were measured by qRT-PCR, β-actin was used as a control. Data are presented as the mean ± SD. *p < 0.05, **p < 0.01, ***p < 0.001.

MS023 stands out as a powerful, selective, and cell-permeable inhibitor of the enzymatic activity of human type I PRMTs. It can effectively inhibit the enzymatic activity of PRMT1 within the cells [23]. As shown in Fig. 7A, B, MS023 treatment significantly reduced the methylation level of GRP94 and the total level of GRP94 protein. Western blotting and IF experiments demonstrated that MS023 inhibited the expression of fibrosis-related proteins induced by TGF-β1, while the MS023 has no significant effect on the quiescent state of LX2 cells (Fig. 7C–E). Furthermore, MS023 inhibited TGF-β1-induced cell migration (Fig. 7F, G) and rescued the consumption of intracellular lipids (Fig. 7H, I) by TGF-β1. We found that after the addition of MS023, the activation of the PERK-eIF2α-ATF4 pathway induced by TGF-β1 was inhibited (Fig. 7J). Due to the expression level of ATF4 was inhibited by MS023, the mRNA levels of the genes regulated by ATF4 (*ACTA2* and *COL1A1*) also decreased significantly (Fig. 7K). These data indicate that MS023 reduces the protein level of GRP94 by inhibiting its methylation, thereby suppressing ER stress and ultimately inhibiting the activation of HSCs.

### MS023 ameliorates CCl_4_-induced or CDAHFD-induced liver fibrosis in mice

Given that PRMT1 inhibitor MS023 can suppress HSCs activation by inhibiting GRP94 methylation, we hypothesized that MS023 might exhibit anti-fibrotic potential. To verify this hypothesis, we established mouse liver fibrosis models using two distinct methods that had previously been validated: CCl_4_ chemical induction and CDAHFD dietary induction. In the two models, we systematically evaluated the impact of MS023 on liver fibrosis.

**Fig. 8:**
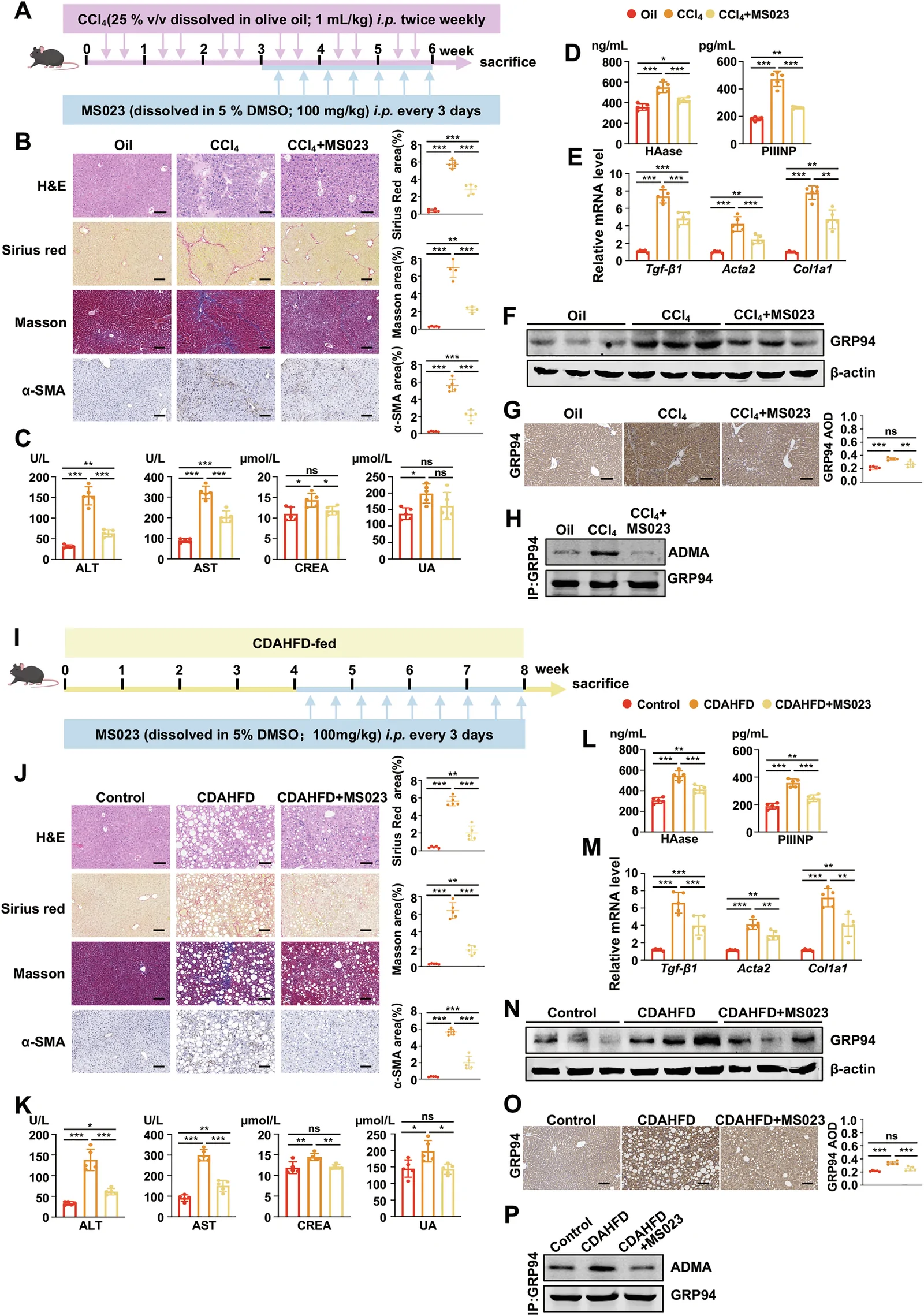
MS023 ameliorates CCl_4_-induced or CDAHFD-induced liver fibrosis in mice. **A**–**E** MS023 attenuates CCl_4_-induced liver fibrosis in mice. **A** Schematic illustration of the experimental design for MS023 treatment in the CCl_4_-induced fibrosis mouse model (*n* = 5). **B** Representative images of hematoxylin and eosin (H&E), Masson’s trichrome, and Sirius Red staining, as well as immunohistochemistry (IHC) for α-SMA, in liver sections from CCl_4_-induced mice treated with or without MS023. Quantification of positive areas is shown on the right. Scale bar, 100 μm. **C** Serum levels of alanine aminotransferase (ALT), aspartate aminotransferase (AST), urea, creatinine (CREA), and uric acid (UA) in the indicated treatment groups. **D** Serum levels of hyaluronidase (HAase) and N-terminal peptide of type III procollagen (PIIINP) measured by ELISA in the indicated treatment groups. **E** MS023 reduces the fibrogenic gene mRNA level of GRP94 in CCl_4_-induced liver fibrosis mouse model. qRT-PCR analysis of fibrogenic gene expression. **F**–**H** MS023 reduces the protein level and methylation level of GRP94 in liver of CCl_4_-induced mouse model. **F** Western blot was performed to determine the expression of GRP94 in liver of mice. **G** Representative IHC images of GRP94 in the liver sections and quantification of average optical density (AOD). Scale bar, 100 μm. **H** The mouse liver tissue protein lysate was incubated with an anti-GRP94 antibody, followed by Western blotting using the anti-ADMA antibody. **I**–**M** MS023 attenuates CDAHFD-induced liver fibrosis in mice. **I** Schematic illustration of the experimental design for MS023 treated in the CDAHFD-induced fibrosis mouse model (*n* = 5). **J** Representative images of H&E, Masson’s trichrome, and Sirius Red staining, as well as IHC for α-SMA, in liver sections from CDAHFD-induced mice treated with or without MS023. Quantification of positive areas is shown on the right. Scale bar, 100 μm. **K** Serum levels of ALT, AST, CREA, and UA in the indicated treatment groups. **L** Serum levels of HAase and PIIINP measured by ELISA in the indicated treatment groups. **M** MS023 reduces the fibrogenic gene mRNA levels in CDAHFD-induced liver fibrosis mouse model. qRT-PCR analysis of fibrogenic gene expression. **N**–**P** MS023 reduces GRP94 protein level and methylation level in CDAHFD-induced fibrosis mice. **N** Western blot was performed to determine the expression of GRP94 in liver of mice. **O** Representative IHC images of GRP94 in liver sections and quantification of AOD. Scale bar Scale bar, 100 μm. **P** The mouse liver tissue protein lysate was incubated with an anti-GRP94 antibody, followed by Western blotting using the anti-ADMA antibody. Data are presented as the mean ± SD. *p < 0.05, **p < 0.01, ***p < 0.001.

In the CCl_4_-induced mouse liver fibrosis model (Fig. 8A), the liver tissues in CCl_4_ group exhibited marked fibrosis, inflammation, severe liver injury and collagen deposition. Significantly, MS023 attenuated liver injury and reduced the area of collagen deposition in the liver of CCl_4_-induced mice, as evidenced by H&E staining, Masson staining, Sirius red and α-SMA staining (Fig. 8B). Analysis of ALT, AST, CREA and UA levels in the mouse serum indicated that treatment with MS023 resulted in the restoration of liver and kidney function in liver fibrosis mice (Fig. 8C). After treatment with MS023, the levels of HAase and PNIIIP in the mouse serum were significantly decreased (Fig. 8D). The qRT-PCR analysis demonstrated that MS023 reduced the expression of key genes related to liver fibrosis (Fig. 8E). The results of Western blotting (Fig. 8F) and immunohistochemistry (Fig. 8G) indicated that MS023 could reduce the expression of GRP94 protein in fibrotic liver tissues induced by CCl_4_. The total protein of mouse liver tissue was immunoprecipitated using GRP94 antibody. The results showed that MS023 could reduce the methylation level of GRP94 protein in fibrotic liver tissue induced by CCl_4_ (Fig. 8H).

In the CDAHFD-induced liver fibrosis model (Fig. 8I), a distinct anti-fibrotic effect was also observed following MS023 intervention. Histopathological analysis revealed significant aggravation of liver injury, hepatic steatosis, inflammatory infiltration, and fibrosis-related collagen deposition in CDAHFD-induced mice, whereas these pathological alterations were notably ameliorated by MS023 intervention (Fig. 8J). Serological analyses further confirmed the MS023 reduced liver and kidney damage in mice (Fig. 8K), and downregulated the serum fibrosis markers (Fig. 8L). qRT-PCR assay demonstrated that MS023 downregulated the expression of key genes associated with liver fibrosis (Fig. 8M). As shown in Fig. 8N, O, the experiments of Western blotting and immunohistochemistry indicated that MS023 also reduced the expression of GRP94 protein in CDAHFD-induced fibrotic liver tissues. The results of immunoprecipitation indicating that MS023 can reduce the methylation level of GRP94 protein in fibrotic liver tissues induced by CDAHFD (Fig. 8P).

Collectively, these findings indicate that MS023 inhibits GRP94 methylation and reduces the level of GRP94 protein in the liver, exerting a significant anti-fibrotic effect in both CCl_4_-induced and CDAHFD-induced mouse liver fibrosis models.

## Discussion

Liver fibrosis represents the defining, reversible transition from chronic liver injury to end-stage disease. Clinically, the imperative is to intervene at this pivotal window—where timely action can halt or reverse its course and prevent the cascade toward cirrhosis and its complications [37]. Currently, targeted therapeutic strategies focusing on inhibiting HSC activation represent the core areas of anti-fibrosis research, has significant clinical implications for the development of improved treatments for fibrotic liver diseases [3, 38]. Our study uncovers a previously unrecognized regulatory axis in liver fibrogenesis, wherein PRMT1-mediated methylation of the ER chaperone GRP94 serves as a critical amplifier of HSC activation. This finding positions the crosstalk between ER stress and protein arginine methylation as a central node in the pathogenesis of liver fibrosis. We demonstrate that GRP94, whose expression is elevated in liver fibrosis, promotes ER stress, robustly enhances the activation of HSCs. The methylation of GRP94 catalyzed by PRMT1 enhances the stability of GRP94, allowing it to be stably and highly expressed in HSCs, thereby promoting the activation of HSCs. MS023, which is a PRMT1 enzyme activity inhibitor, can effectively inhibit the methylation of GRP94, thereby inhibiting ER stress and activation of HSCs. Moreover, its pharmacological inhibitory effect observed in the mouse liver fibrosis model can alleviate the symptoms of fibrosis. A summary model is shown in graphical abstract.

Our study found that in both mouse models of liver fibrosis induced by CCl_4_ (chemical injury) and CDAHFD (metabolic injury), despite their distinct initial mechanisms, significant pathological changes characteristic of liver fibrosis was observed. These were accompanied by aberrant protein accumulation within the ER lumen and an overall increase in protein methylation levels in liver tissue. Despite distinct initial injury mechanisms, both models converged on these shared molecular events, suggesting that disruption of hepatocyte protein homeostasis and subsequent ER stress may represent a common driver of fibrosis across etiologies. Sustained stress not only activates the UPR and drives inflammatory and fibrotic signaling pathways [39] but may also affect the methylation modification status of a range of functional proteins, including those related to ER stress, by regulating methyltransferase activity or substrate accessibility.

Our experimental results indicate that during the process of liver fibrosis, the expression of GRP94, a key chaperone protein involved in ER stress, was significantly upregulated. GRP94 could further enhance ER stress levels through a positive feedback mechanism by stabilizing and activating the PERK-eIF2α-ATF4 pathway, thereby driving the expression of fibrosis-related genes and the activation of HSCs and the expression of fibrosis-related genes. Mechanistically, during the development of liver fibrosis, the ER homeostasis in HSCs is disrupted [11, 37, 40], inducing high expression of GRP94. While GRP94 is known as a chaperone responsible for protein folding and clearance of misfolded proteins [41, 42], its expression and secretion have also been directly linked to TGF-β1 production and fibrosis initiation [43]. Here, we verified that the highly expressed GRP94 is no longer merely a marker of ER stress. Instead, by engaging with the specific PERK-eIF2α-ATF4 signaling pathway, it establishes a positive feedback vicious cycle mechanism of ER stress, which leads to the continuous intensification of ER stress and prompts HSCs to maintain an activated pro-fibrotic phenotype. This provides a molecular explanation for the persistence and intensification of ER stress in liver fibrosis, revealing a key amplification mechanism that converts initial stress signals into long-term, stable pro-fibrotic outputs, and offers new perspectives for understanding the long-term maintenance and malignant progression of liver fibrosis.

Furthermore, this study revealed that GRP94 undergoes methylation modification. Based on our previous research on arginine methyltransferase in our group [34], we further determined that PRMT1 is the enzyme that catalyzes the methylation of GRP94. This modification inhibits GRP94 degradation via the ubiquitin–proteasome pathway, enhancing its protein stability and leading to sustained high expression in HSCs. The stabilized GRP94 protein not only acts as an effector molecule of ER stress but may also further exacerbate ER dysfunction, creating a vicious cycle that promotes the progression of fibrosis. Previous studies on liver fibrosis have shown that PRMT1 can participate in the progression of metabolic-associated fatty liver disease (MASLD) by activating HSC [22]. PRMT1 regulates the pro-inflammatory NF-κB and pro-fibrotic TGF-β1 signaling pathways, influencing the expression of fibrosis-related genes, thereby affecting the activation degree of hepatic stellate cells, lipid and glucose metabolism, and ultimately influencing the occurrence and development of liver fibrosis [25, 44]. These researches align with our study that PRMT1, by methylating GRP94, directly amplifies ER stress signaling, thereby driving HSC activation and liver fibrotic development. This discovery establishes a direct functional link between PRMT1 activity, post-translational regulation of GRP94, and ER stress amplification, defining a novel ‘PRMT1–GRP94–ER stress’ axis that deepens understanding of protein methylation in liver fibrosis and offers new potential therapeutic targets.

Our research indicates that the methylated GRP94 has a stronger ability to promote the activation of HSCs compared to its wild-type form. By treating GRP94 with the PRMT1 inhibitor MS023, it can effectively inhibit its methylation and protein expression, thereby significantly slowing down the progression of liver fibrosis in HSCs and in the CCl_4_ and CDAHFD mouse models. Moreover, this treatment can also alleviate the liver and kidney damage in the model mice. The fundamental cause of this phenomenon may lie in the fact that methylation modification inhibits the ubiquitin-mediated degradation pathway of GRP94, thereby significantly enhancing its protein stability and prolonging its half-life in the cell. The more stable GRP94 protein can continuously activate the ER stress signaling pathway, PERK-eIF2α-ATF4, thereby establishing a continuously expanding positive feedback loop, maintaining the long-term activation state of HSCs. It should be noted that although the MS023 used in this study is not a specific inhibitor of PRMT1, but a broad-spectrum inhibitor targeting type I PRMTs (including PRMT1, 4, and 6), the experimental evidence has confirmed its definite and effective inhibition of GRP94 methylation and the subsequent pro-fibrotic pathways. This indirectly indicates that inhibiting the methylation of GRP94 methylation itself is a key step in anti-fibrotic therapy. This discovery has important theoretical and translational value. From a theoretical perspective, it establishes a direct connection between protein methylation modification and the strength and duration regulation of the ER stress signal, deepening the understanding of the self-maintaining mechanism of fibrosis. From a translational medicine perspective, although MS023 used in this study does not have a specific targeting limitation, but our findings provide key theoretical validation for the development of new therapeutic approaches targeting GRP94 methylation. It should be noted that the broad-spectrum action of MS023 means that its anti-fibrotic effect may also benefit from the inhibition of the methylation of other key protein molecules. These unidentified substrates may jointly promote the final improvement of the pathological phenotype. This insight reveals the complexity of the PRMT-mediated methylation regulatory network. Future research can adopt techniques such as methyl proteomics to systematically screen and verify other functional proteins regulated by PRMTs that play a key role in liver fibrosis, aiming to draw a more comprehensive pathogenic network. Based on this, subsequent research will focus on screening or designing more specific PRMT1 inhibitors, aiming to maximize the anti-fibrotic efficacy while minimizing potential non-targeting effects, thereby promoting the development of more clinically applicable targeted treatment strategies for liver fibrosis.

In conclusion, our study systematically revealed the crucial synergistic effect of ER stress and protein arginine methylation in liver fibrosis, and elucidated that PRMT1 methylates the ER chaperone protein GRP94, enhancing its stability and forming persistent ER stress, which promotes the activation of HSCs and thereby drives the progression of liver fibrosis. The inhibitor MS023 of PRMT1 demonstrated effective alleviation of liver fibrosis in experiments, which provided important preclinical evidence for it as a potential targeted therapeutic strategy for liver fibrosis and laid the theoretical foundation for the development of related anti-fibrotic drugs.

## Supplementary information

Supplementary Figure

Supplementary materials and methods

Supplementary Table1

Supplementary Table2

Original western blots

## Data Availability

All data and materials are available in the main text and supplementary information.
