# Supplementary material for: Methylated GRP94 promotes liver fibrosis progression by enhancing endoplasmic reticulum stress
Source: Cell Death Dis. 2026 Aug 5;17(1):910. doi: 10.1038/s41419-026-09159-1 (PMC13713989; doi:10.1038/s41419-026-09159-1)
Supplement: Supplementary file 1 [file 41419_2026_9159_MOESM5_ESM.pdf]

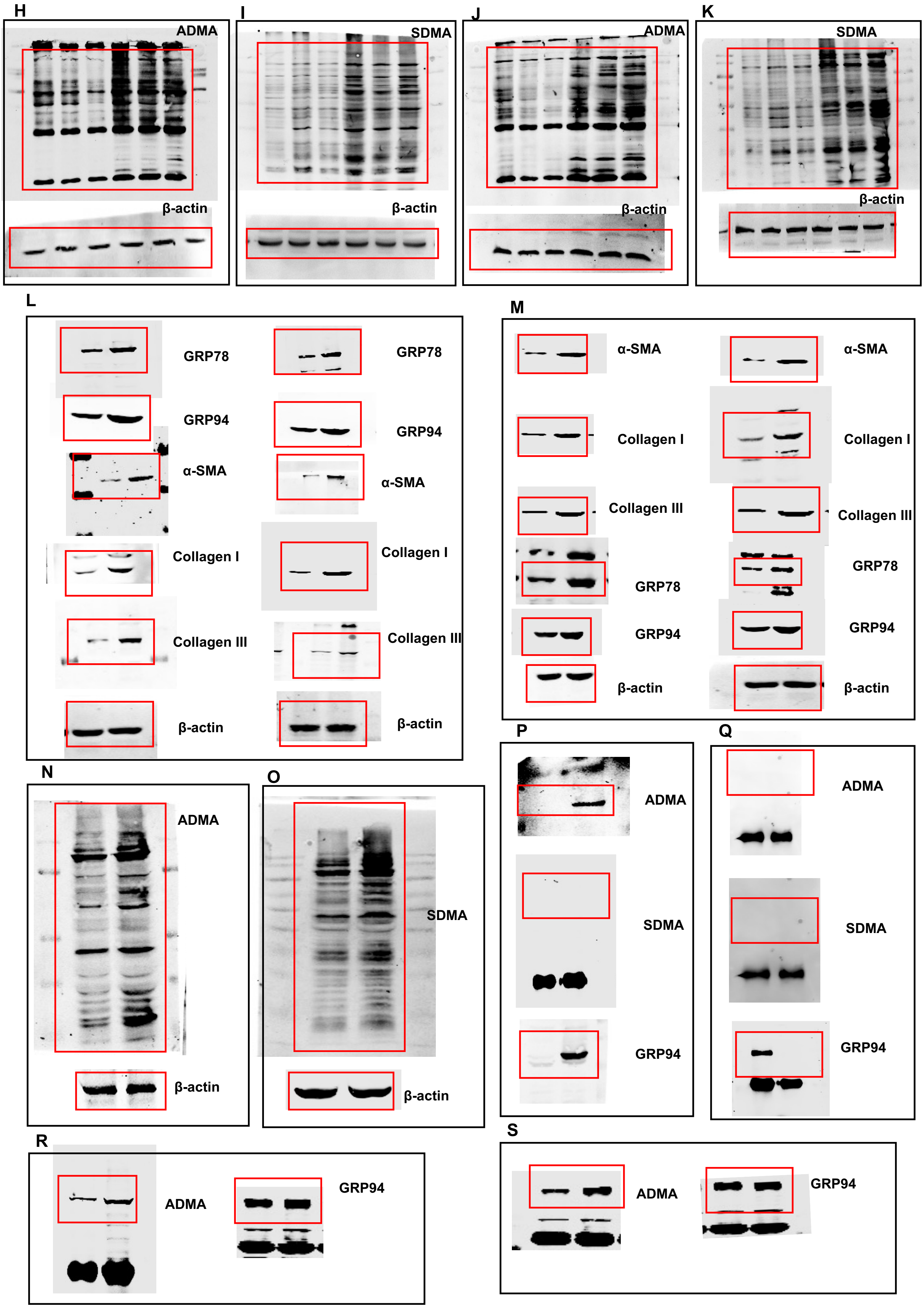
H
ADMA
β-actin
I
SDMA
β-actin
J
ADMA
β-actin
K
SDMA
β-actin
L
GRP78
GRP94
α-SMA
Collagen I
Collagen III
β-actin
GRP78
GRP94
α-SMA
Collagen I
Collagen III
β-actin
M
α-SMA
Collagen I
Collagen III
GRP78
GRP94
β-actin
α-SMA
Collagen I
Collagen III
GRP78
GRP94
β-actin
N
ADMA
β-actin
O
SDMA
β-actin
P
ADMA
SDMA
GRP94
Q
ADMA
SDMA
GRP94
R
ADMA
GRP94
S
ADMA
GRP94

Figure1

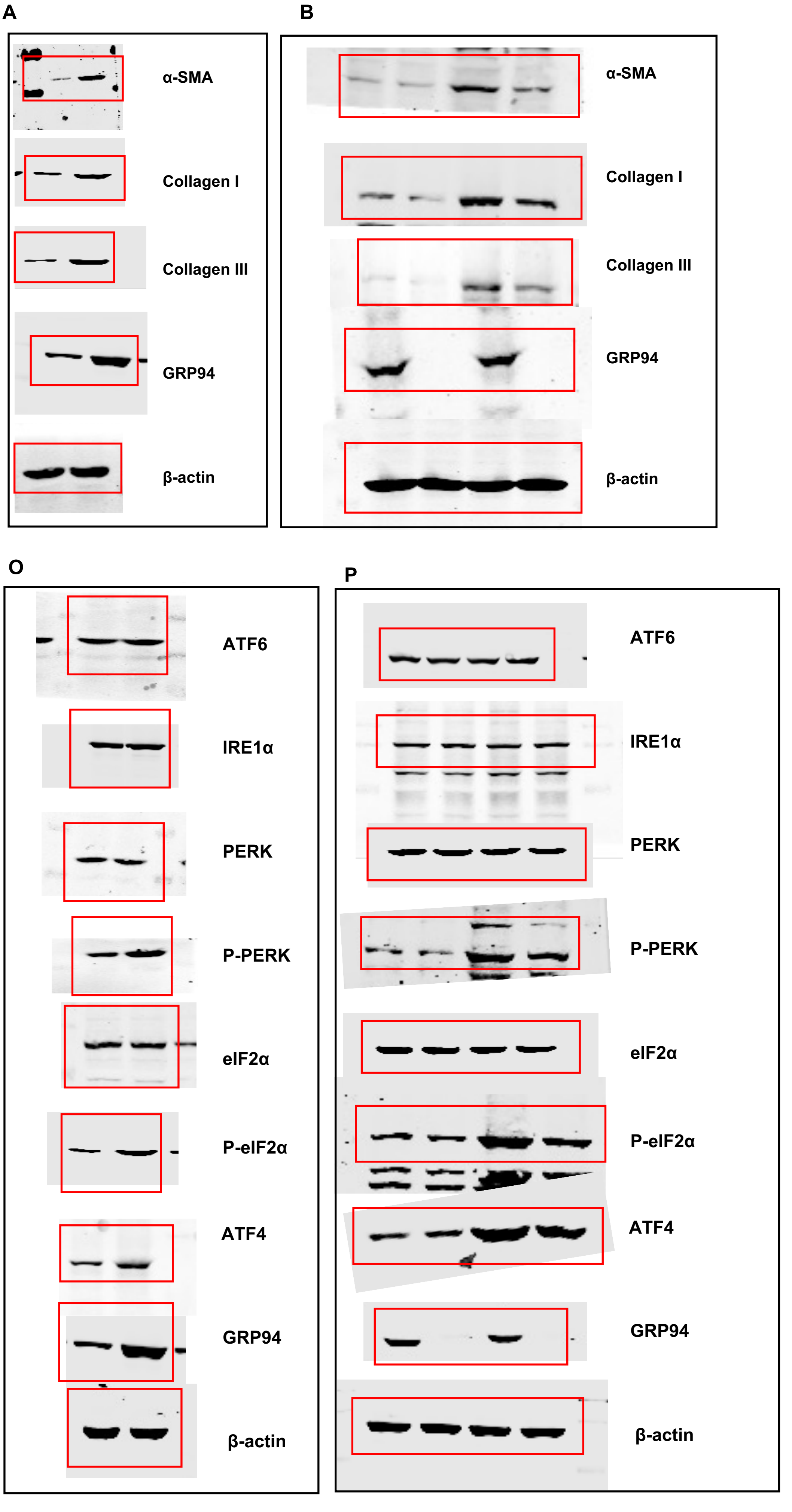
A
α-SMA
Collagen I
Collagen III
GRP94
β-actin
B
α-SMA
Collagen I
Collagen III
GRP94
β-actin
O
ATF6
IRE1α
PERK
P-PERK
eIF2α
P-eIF2α
ATF4
GRP94
β-actin
P
ATF6
IRE1α
PERK
P-PERK
eIF2α
P-eIF2α
ATF4
GRP94
β-actin

Figure2

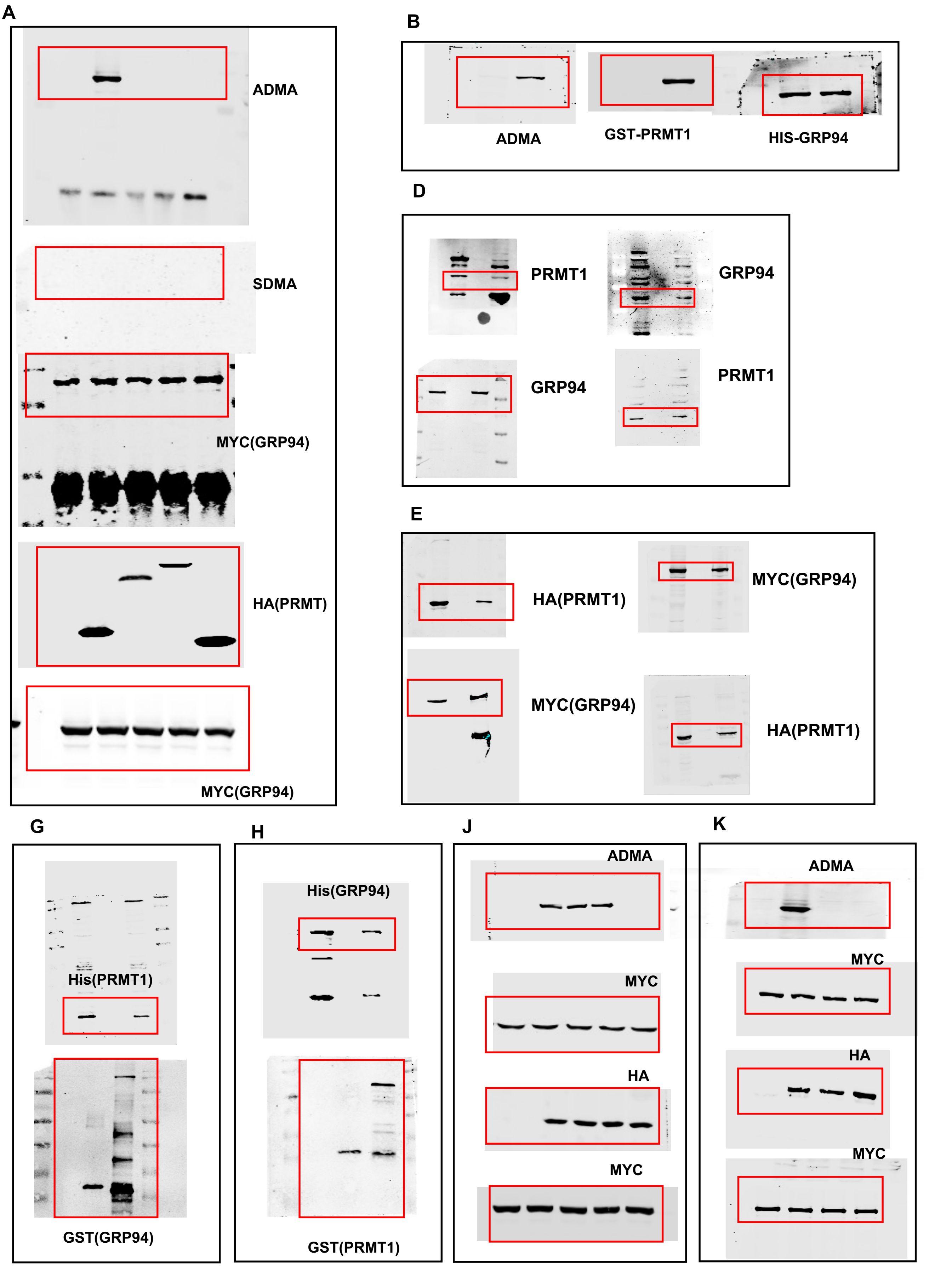
A
ADMA
SDMA
MYC(GRP94)
HA(PRMT)
MYC(GRP94)
B
ADMA
GST-PRMT1
HIS-GRP94
D
PRMT1
GRP94
GRP94
PRMT1
E
HA(PRMT1)
MYC(GRP94)
MYC(GRP94)
HA(PRMT1)
G
His(PRMT1)
GST(GRP94)
H
His(GRP94)
GST(PRMT1)
J
ADMA
MYC
HA
MYC
K
ADMA
MYC
HA
MYC

Figure3

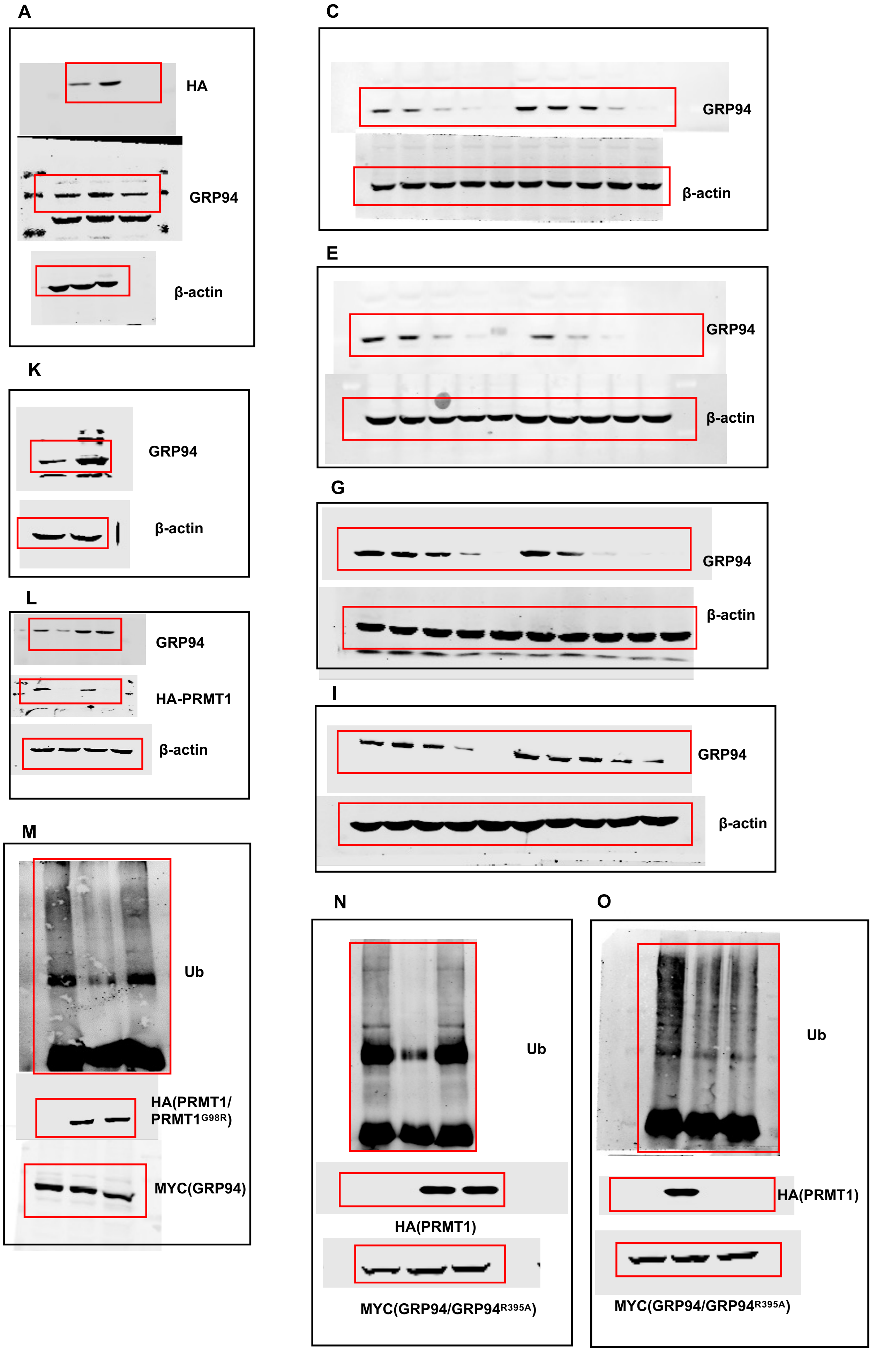
A
HA
GRP94
β-actin
C
GRP94
β-actin
E
GRP94
β-actin
K
GRP94
β-actin
G
GRP94
β-actin
L
GRP94
HA-PRMT1
β-actin
I
GRP94
β-actin
M
Ub
HA(PRMT1/
PRMT1G98R)
MYC(GRP94)
N
Ub
HA(PRMT1)
MYC(GRP94/GRP94R395A)
O
Ub
HA(PRMT1)
MYC(GRP94/GRP94R395A)

Figure4

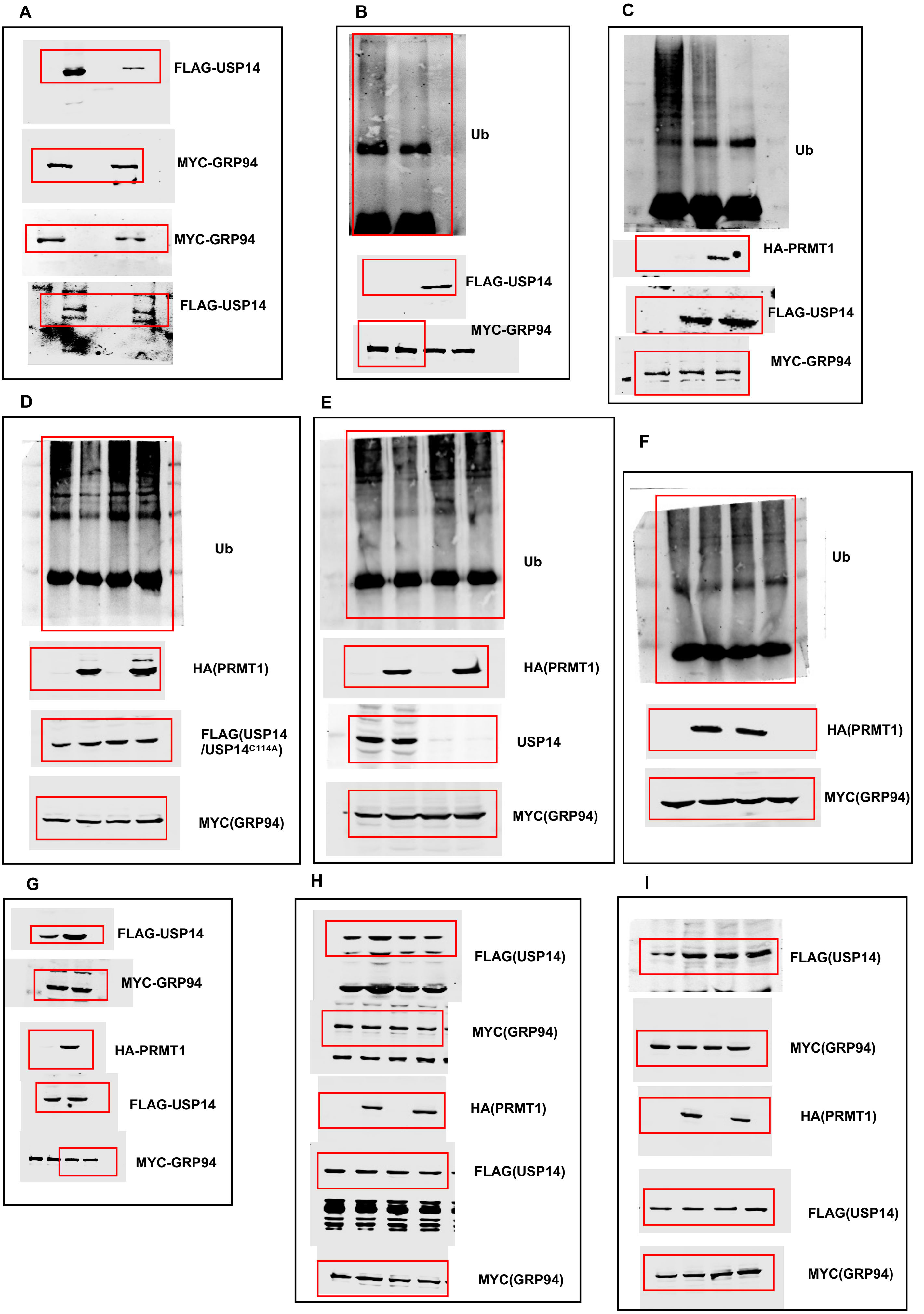
A
FLAG-USP14
MYC-GRP94
MYC-GRP94
FLAG-USP14
B
Ub
FLAG-USP14
MYC-GRP94
C
Ub
HA-PRMT1
FLAG-USP14
MYC-GRP94
D
Ub
HA(PRMT1)
FLAG(USP14
/USP14C114A)
MYC(GRP94)
E
Ub
HA(PRMT1)
USP14
MYC(GRP94)
F
Ub
HA(PRMT1)
MYC(GRP94)
G
FLAG-USP14
MYC-GRP94
HA-PRMT1
FLAG-USP14
MYC-GRP94
H
FLAG(USP14)
MYC(GRP94)
HA(PRMT1)
FLAG(USP14)
MYC(GRP94)
I
FLAG(USP14)
MYC(GRP94)
HA(PRMT1)
FLAG(USP14)
MYC(GRP94)

Figure5

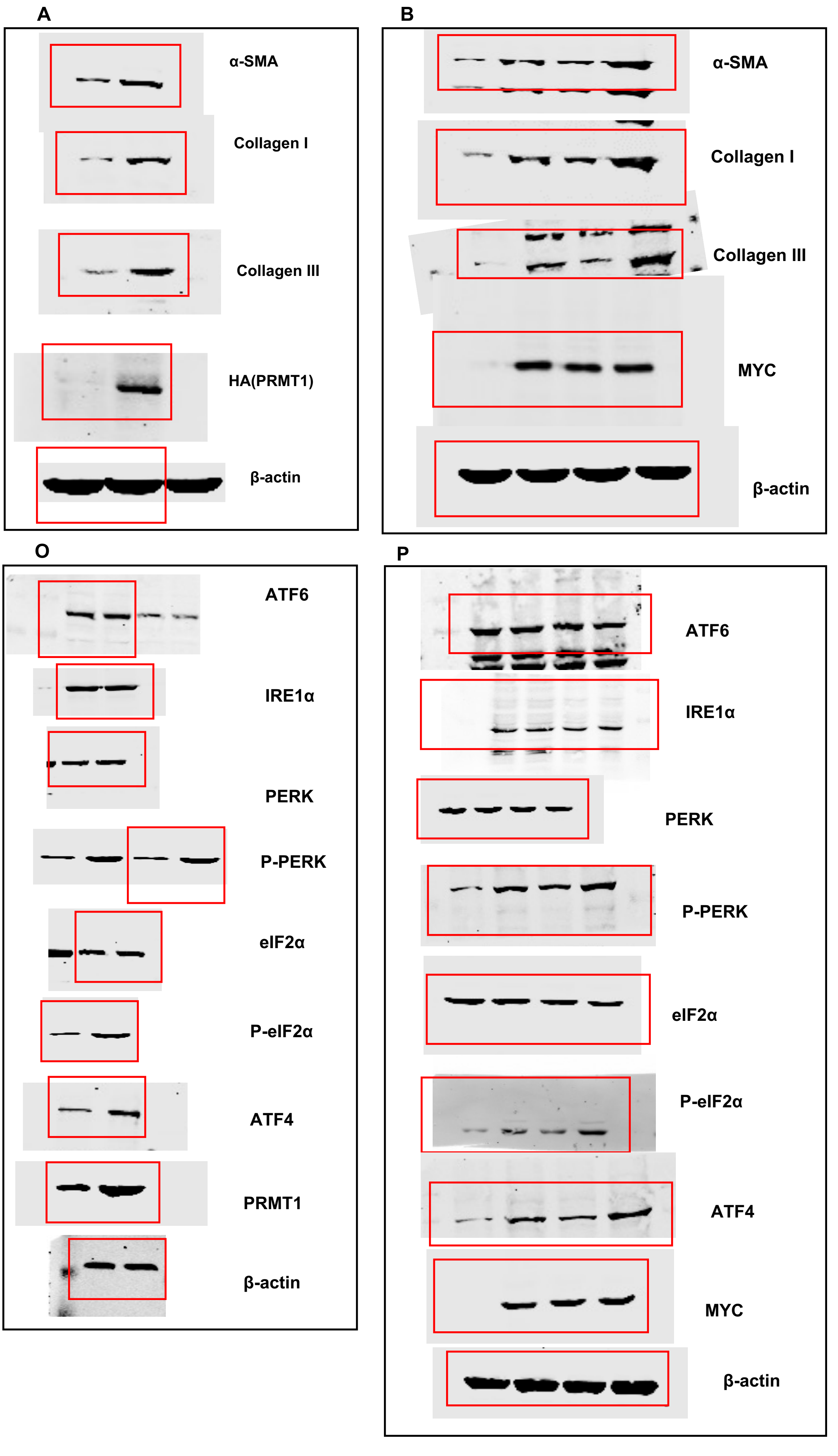
A
α-SMA
Collagen I
Collagen III
HA(PRMT1)
β-actin
B
α-SMA
Collagen I
Collagen III
MYC
β-actin
O
ATF6
IRE1α
PERK
P-PERK
eIF2α
P-eIF2α
ATF4
PRMT1
β-actin
P
ATF6
IRE1α
PERK
P-PERK
eIF2α
P-eIF2α
ATF4
MYC
β-actin

Figure6

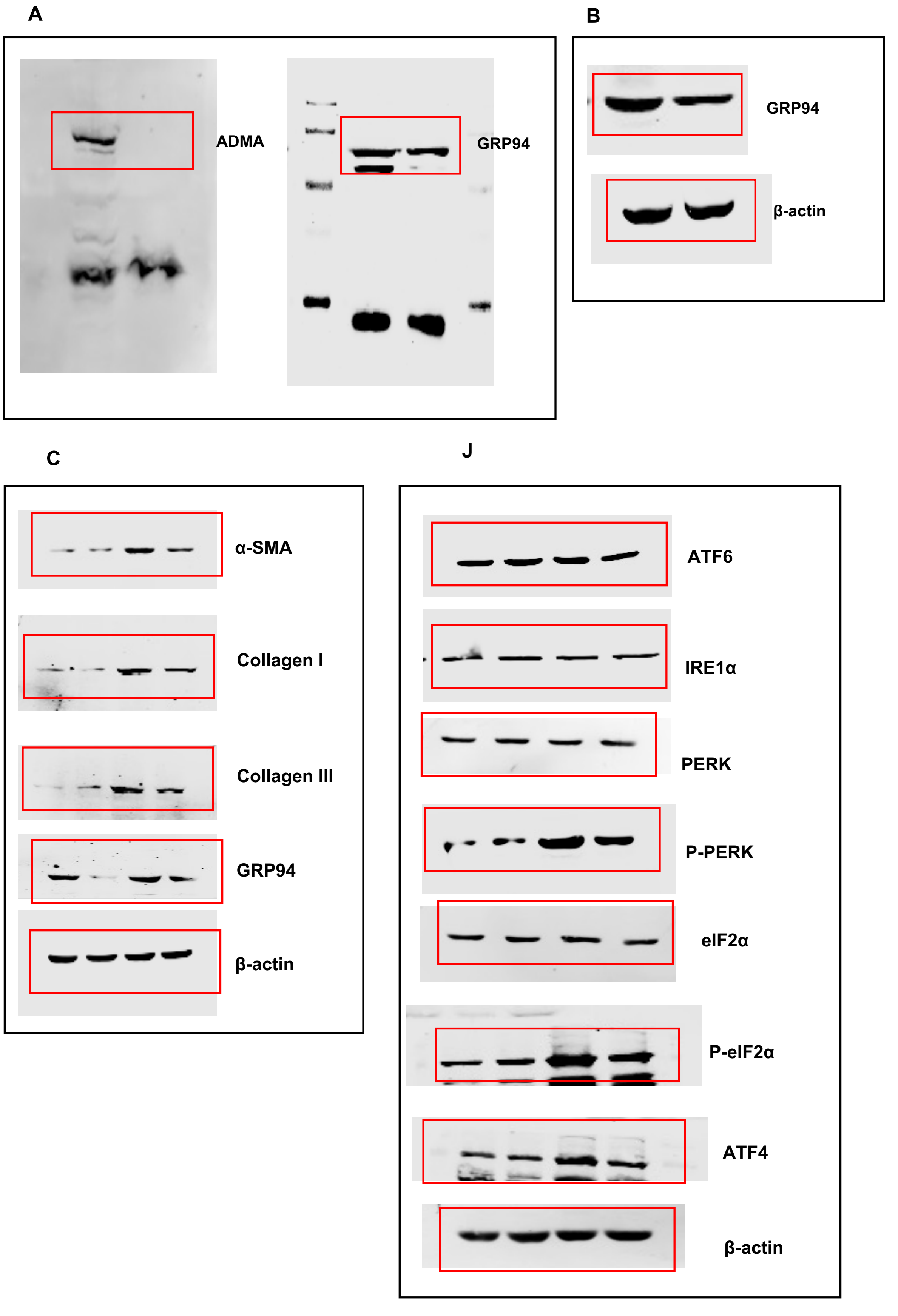
A
ADMA
GRP94
B
GRP94
β-actin
C
α-SMA
Collagen I
Collagen III
GRP94
β-actin
J
ATF6
IRE1α
PERK
P-PERK
eIF2α
P-eIF2α
ATF4
β-actin

Figure7

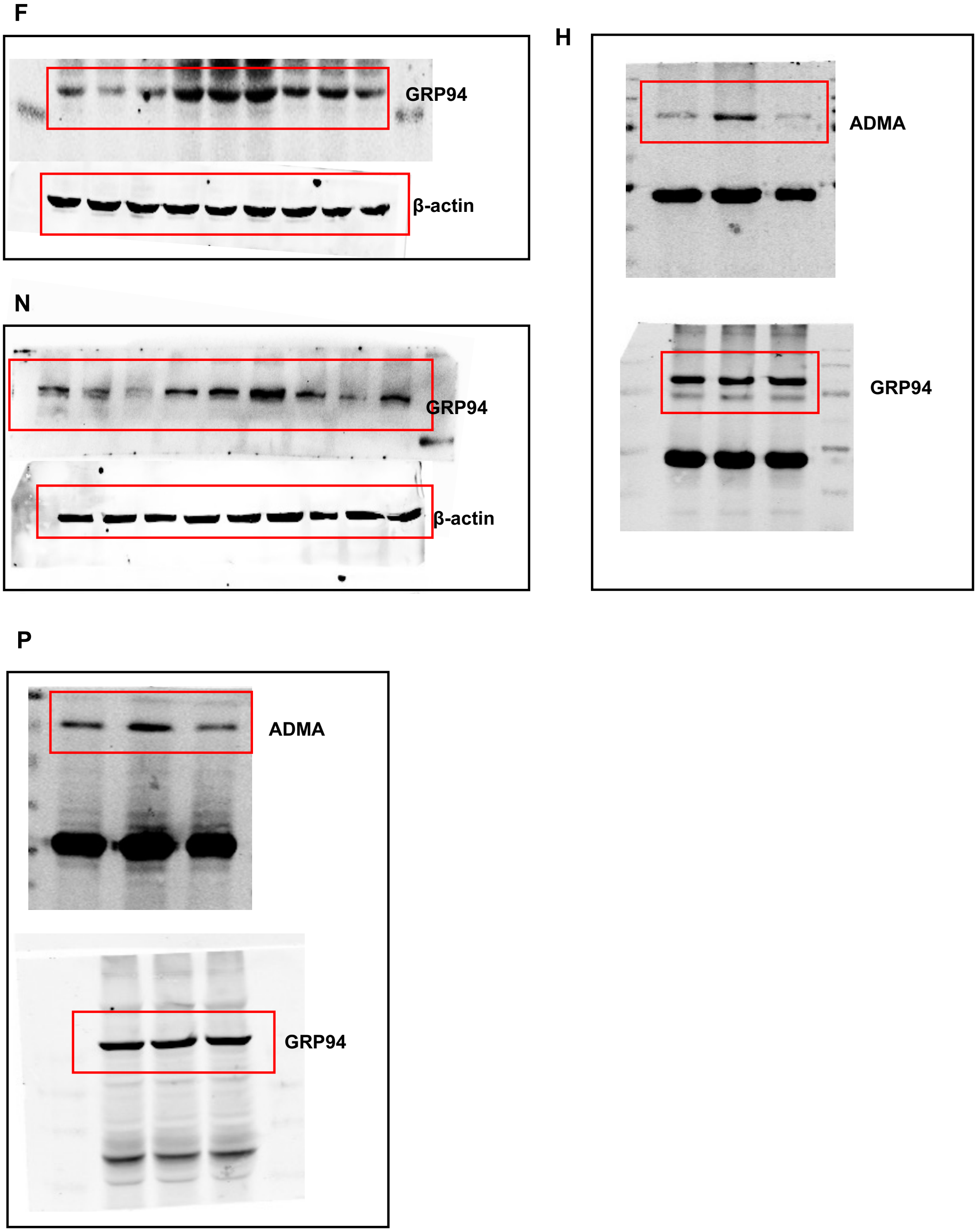

Figure8
