# Supplementary material for: Methylated GRP94 promotes liver fibrosis progression by enhancing endoplasmic reticulum stress
Source: Cell Death Dis. 2026 Aug 5;17(1):910. doi: 10.1038/s41419-026-09159-1 (PMC13713989; doi:10.1038/s41419-026-09159-1)
Supplement: Supplementary file 2 [file 41419_2026_9159_MOESM1_ESM.docx]

**Fig. S1 CCl_4_ or CDAHFD induce liver fibrosis in mice.**

**A-B** Quantification of Sirius Red, Masson, and α-SMA IHC staining in liver sections from **(A)** CCl_4_- or **(B)**CDAHFD-induced fibrotic mice. Scale bar, 100 μm. **C-D** Serum levels of alanine aminotransferase (ALT), aspartate aminotransferase (AST), creatinine (CREA), and uric acid (UA) in the indicated treatment groups. **E-F** Serum levels of hyaluronidase (HAase) and N-terminal peptide of type III procollagen (PIIINP) measured by ELISA in the indicated treatment groups. **G-H** qRT-PCR analysis of fibrogenic gene expression in mouse liver tissues. **I** Heatmap illustrating differentially expressed proteins from the top 20 terms identified by Gene Ontology (GO) Molecular Function enrichment analysis. **J-K** Protein levels of GRP94 and PRMT1 are significantly increased in fibrotic liver tissues from CCl4- or CDAHFD-induced mice. Representative IHC images of GRP94 and PRMT1 in liver sections and quantification of average optical density (AOD). Scale bar, 100 μm. Data are presented as the mean ± SD. *p< 0.05, **p< 0.01, ***p< 0.001.

**Fig. S2**

**A** Fragmentation spectrum of the GRP94 methylated peptide identified by liquid chromatography/tandem mass spectrometry (LC-MS/MS). **B** LX-2 cells were treated with TGF-β1, Tg, or MS023, respectively, and analyzed by LC-MS/MS. Compare the relative abundance of modified peptides in different treatment groups.
