# Supplementary material for: Methylated GRP94 promotes liver fibrosis progression by enhancing endoplasmic reticulum stress
Source: Cell Death Dis. 2026 Aug 5;17(1):910. doi: 10.1038/s41419-026-09159-1 (PMC13713989; doi:10.1038/s41419-026-09159-1)
Supplement: Supplementary file 3 [file 41419_2026_9159_MOESM2_ESM.docx]

**1.Supplementary Materials**

1.1 **Antibodies**

| **Name** | **Citation** | **Supplier** | **Cat no.** | **Clone no.** |
| --- | --- | --- | --- | --- |
| Anti-Beta Actin | PMID: 37098345 | Proteintech | 66009-1-lg | 2D4H5 |
| Anti-ADMA | PMID: 41371957 | Cell Signaling  Technology | 13522T | N/A |
| Anti-SDMA | PMID: 36521495 | Cell Signaling  Technology | A17910 | D6J9M |
| Anti-GRP78 | PMID: 40681898 | Proteintech | 11587-1-AP | N/A |
| Anti-GRP94 | PMID: 39842432 | Abclonal | A0908 | N/A |
| Anti-α-SMA | PMID: 10890559 | BioLegend | 614852 | 1A4 |
| Anti-Collagen1 | PMID: 40404681 | Immunoway | YM8576 | PT0817R |
| Anti-Collagen3 | PMID: 39887250 | Immunoway | YM8111 | PT0180R |
| Anti-ATF6 | PMID: 39002476 | Wanleibio | WL0153 | N/A |
| Anti-IRE1α | PMID: 39754719 | Santa Cruz | sc-390960 | B-12 |
| Anti-PERK | PMID: 38534743 | Santa Cruz | sc-377400 | B-5 |
| Anti-P-PERK | PMID: 39905002 | Wanleibio | WL05295 | T982 |
| Anti-eIF2α | PMID: 35784473 | Immunoway | YM8313 | PT0480R |
| Anti-P-eIF2α | PMID: 35784473 | Immunoway | YM8728 | PT0904R |
| Anti-ATF4 | PMID: 39789010 | Santa Cruz | sc-390063 | B-3 |
| Anti-HA | PMID: 33720291 | BioLegend | 16B12 | AFC-101P |
| Anti-MYC | PMID: 39261729 | Proteintech | 60003-2-Ig | 1A5A2 |
| Anti-His | PMID: 39013469 | Proteintech | 66005-1-Ig | 1B7G5 |
| Anti-FLAG | PMID: 25697406 | Sigma | F1804 | M2 |
| Anti-GST | PMID: 39013469 | Proteintech | 10000-0-AP | N/A |
| Anti-Ub | PMID: 38167292 | Proteintech | 10201-2-AP | N/A |

1.2 **Cell lines**

| **Name** | **Citation** | **Supplier** | **Cat no.** | **Passage no.** | **Authentication test method** |
| --- | --- | --- | --- | --- | --- |
| LX-2 cells | PMID: 24116068 | Cell Bank of Chinese Science Academy | SCSP-527 | 6-8 | STR |
| HEK293T cells | PMID: 34038747 | Cell Bank of Chinese Science Academy | SCSP-502 | 6-8 | STR |
| Primary Human Hepatic Stellate Cells | PMID: 36376267 | Procell | CP-H046 | 1-3 | STR |

1.3 **Organisms**

| **Name** | **Citation** | **Supplier** | **Strain** | **Sex** | **Age** | **Overall n number** |
| --- | --- | --- | --- | --- | --- | --- |
| Wildtype C57BL/6J | N/A | Peking University Health Science Animal Center | C57BL/6J | Male | 6-8  weeks | 60 |

1.4 **Sequence based reagents**

| **Name** | **Sequence** | **Supplier** |
| --- | --- | --- |
| RT-PCR primer Human *GRP94* | Forward: GGATGGTCTGGCAACATGGA  Reverse: CCGAAGCGTTGCTGTTTCAA | Tsingke |
| RT-PCR primer Human *COL1A1* | Forward: GACAGGCGAACAAGGTGACAGAG  Reverse: CAGGAGAACCAGGAGAACCAGGAG | Tsingke |
| RT-PCR primer Human *ACTA2* | Forward: CGTGGCTATTCCTTCGTGACTACTG  Reverse: CGTCAGGCAGTTCGTAGCTCTTC | Tsingke |
| RT-PCR primer Mouse *Tgf-β1* | Forward: ACCAAGGAGACGGAATAC  Reverse: TGTGGAGCTGAAGCAATA | Tsingke |
| RT-PCR primer Mouse *Col1a1* | Forward: CCTAATGCTGCCTTTTCTGC  Reverse: ATGTCCCAGCAGGATTTGAG | Tsingke |
| RT-PCR primer Mouse *Acta2* | Forward: TCAGGGAGTAATGGTTGGAATG  Reverse: GTGTCGGATGCTCTTCAGG | Tsingke |

1.5 **Deposited data**

| **Name of repository** | **Identifier** | **Link** |
| --- | --- | --- |
| Data-Independent Acquisition Proteomics | PXD075920 | http://proteomecentral.org |

1.6 **Other (e.g. drugs, proteins)**

| TGF-β1 | SinoBiology | Cat#10804-HNAC |
| --- | --- | --- |
| Thapsigargin (Tg) | MedChemExpress | Cat# HY-13433 |
| CHX | MedChemExpress | Cat# HY-12320 |
| CCl_4_ | MedChemExpress | Cat# HY-Y0298 |
| CDAHFD | Research Diets | Cat# A06071302 |
| MG132 | MedChemExpress | Cat# HY-13259 |
| MS023 | MedChemExpress | Cat# HY-19615 |

**2.Supplementary methods**

**2.1 DIA proteomics**

We employed two complementary experimental mouse models: one was treated with carbon tetrachloride (CCl_4)_, and the other was fed a high-fat diet lacking choline and containing L-amino acids (CDAHFD). After the experiment, the liver tissues of the mice were collected. The reaction solution (1% SDC/100 mM Tris-HCl, pH=8.5/10 mM TCEP/40 mM CAA) was added to the sample and incubated at 95°C for 10 min to complete protein denaturation, reduction and alkylation. The resulting mixture was centrifuged and then the supernatant was diluted with equal volume of ddH_2_O. Trypsin was added at a ratio of 1:50 (enzyme: protein, w/w) for overnight digestion at 37°C. The next day, TFA was used to bring the pH down to 6.0 to end the digestion. After centrifugation (12000 g, 15 min), the supernatant was subjected to peptide purification using a self-made SDB-RPS desalting column. The peptide eluate was vacuum dried and stored at -20°C for later use.

Informatics protocol: Samples were analyzed on an UltiMate 3000 RSLCnano system coupled on-line with Q Exactive HF mass spectrometer through a Nanospray Flex ion source (Thermo). Peptide samples were injected into a C18 Trap column (75 µm*2 cm, 3 µm particle size, 100 Å pore size, Thermo), and separated in a reversed-phase C18 analytical column packed in-house with ReproSil-Pur C18-AQ resin (75 µm*25 cm, 1.9 µm particle size, 100 Å pore size). Mobile phase A (0.1% formic acid, 3% DMSO, 97% H2O) and mobile phase B (0.1% formic acid, 3% DMSO, 97% ACN) were used to establish the seperation gradient at a flow rate of 300 nL/min. To acquire MS data, DIA scan mode was performed with one full MS event followed by 30 MS/MS windows in one cycle. AGC Target value for the full MS scan was 3E6 charges in the 350-1250 m/z range with a maximum injection time of 30 ms and a resolution of 60,000 at m/z 200. DIA MS/MS scans were acquired at a resolution of 15,000 at m/z 200 with an AGC target value of 1E6, a maximum injection time of 50 ms, and fixed first mass of 120 Th. Fragmentation of precursor ions was performed by higher-energy collisional dissociation (HCD) with a normalized collision energy of 28.

DIA raw data were analyzed with DIA-NN (DIA-NN-v1.8.1) in library-free mode. Spectra files were searched against Human database (2025-01-13,20421 entries). Search parameters were mainly default and set as follows: Precursor ion generation options were enabled for in silico-predicted spectral library generation; Trypsin/P with maximum 1 missed cleavage; Carbamidomethyl on C as fixed modification; Oxidation on M and protein N-terminal acetylation as variable modifications; the mass and MS1 accuracy was set to 15 ppm; MBR was enabled; Heuristic protein inference was enabled; Precursor FDR was set to 1% for reliable identifications. Protein intensities were normalized with MaxLFQ algorithm.

Proteins with a fold change > 4 between bait IP and control were screened out as interactors of the bait protein. GO annotation files (release in July 2020) were downloaded from the Gene Ontology Consortium Web site (http://www.geneontology.org/). GO terms pathways with a p value < 0.05 were statistically enriched among the interactors.

**2.2 LC-MS/MS**

The cell sample was mashed with a glass rod, followed by ddH_2_O, decolorizing solution (Coomassie staining: 50% ACN/100 mM NH_4_HCO_3_, pH=8.0; Silver staining: 30 mM K_3_Fe(CN)_6_/100 mM Na2S2O3) and ACN for decolorization. The sample was vortexed for 5 min, centrifuged and the supernatant was discarded. Protein reduction and alkylation were conducted with TCEP and CAA at 60°C for 30 min. The sample was vortexed for 5 min with ACN addition, centrifuged to discard the supernatant and then vacuum dried. An appropriate amount of trypsin was added according to the sample volume for overnight digestion at 37°C. The next day, the peptide extraction solution (60% ACN/5% formic acid) was added and then the mixture was sonicated for 10 min, centrifuged to remove the supernatant and vacuum dried. The C18 desalting column was used for desaltlination treatment. The sample was vacuum dried and stored at -20°C for later use.

All samples were analyzed on timsTOF Pro (Bruker Daltonics), a hybrid trapped ion mobility spectrometer (TIMS) quadrupole time-of-flight mass spectrometer. An UltiMate 3000 RSLCnano system (Thermo) was coupled to timsTOF Pro with a CaptiveSpray nano ion source (Bruker Daltonics). Peptide samples were injected into a C18 Trap column (75 µm*2 cm, 3 µm particle size, 100 Å pore size, Thermo), and separated in a reversed-phase C18 analytical column (75 µm*15 cm, 1.7 µm particle size, 100 Å pore size, IonOpticks). Mobile phase A (0.1% formic acid in water) and mobile phase B (0.1% formic acid in ACN) were used to establish the seperation gradient at a flow rate of 300 nL/min. The MS data acquisition was performed in PASEF mode. The capillary voltage was set to 1500 V. The MS and MS/MS spectra were acquired from 100 to 1700 m/z. The ion mobility was scanned from 0.75 to 1.4 Vs/cm2. The accumulation time and ramp time were set to 166 ms. The acquisition cycle of 1.88 s comprised one full MS scan and 10 PASEF MS/MS scans. Singly charged precursors were filtered out by ion mobility, and only precursor signals over an intensity threshold of 1000 were picked for fragmentation. Target intensity was set to 20000. Precursors were dynamically excluded for 0.4 min. The quardrupole isolation width was set as 2.0 Da at m/z 700, 3.0 Da at m/z 800. The collision energy was ramped linearly as a function of the mobility from 59 eV at 1/K0=1.6 Vs/cm2 to 20 eV at 1/K0=0.6 Vs/cm2.

MS raw data were analyzed with MaxQuant (2.2.0.0) using the Andromeda database search algorithm. Spectra files were searched against the Human protein sequence database downloaded from Uniprot (20250113)using the following parameters: variable modifications were set as Carbamidomethyl (C)-57.021464, Oxidation(M)-15.994915, Methyl(R)-14.015650, Dimethyl(R)-28.031300, fixed modification was set as Carbamidomethyl (C)-57.021464; Enzyme digestion specificity was set to Trypsin/P with maximum 2 missed cleavages; Peptide mass tolerance in first search and main search were set to 20 ppm and 10 ppm; Fragment match tolerance was set to 20 ppm.

Proteins could not be distinguished on the basis of unique peptides were merged by MaxQuant into one protein group. Search results were filtered with 1% FDR at both peptide and protein levels.
